# Hybrid deep learning optimization for smart agriculture: Dipper throated optimization and polar rose search applied to water quality prediction

**DOI:** 10.1371/journal.pone.0327230

**Published:** 2025-07-21

**Authors:** Amal H. Alharbi, Faris H. Rizk, Khaled Sh. Gaber, Marwa M. Eid, El-Sayed M. El-kenawy, Ehsan Khodadadi, Nima Khodadadi

**Affiliations:** 1 Department of Computer Sciences, College of Computer and Information Sciences, Princess Nourah Bint Abdulrahman University, Riyadh, Saudi Arabia; 2 Department of Communications and Electronics, Delta Higher Institute of Engineering and Technology, Mansoura, Egypt; 3 Computer Science and Intelligent Systems Research Center, Blacksburg, Virginia, United States of America; 4 Faculty of Artificial Intelligence, Delta University for Science and Technology, Mansoura, Egypt; 5 Jadara University Research Center, Jadara University, Irbid, Jordan; 6 Department of Programming, School of Information and Communications Technology (ICT), Bahrain Polytechnic, Isa Town, Bahrain; 7 Applied Science Research Center, Applied Science Private University, Amman, Jordan; 8 Department of Chemistry and Biochemistry, University of Arkansas, Fayetteville, Arkansas, United States of America; 9 Department of Civil and Architectural Engineering, University of Miami, Coral Gables, Florida, United States of America; Swedish Meteorological and Hydrological Institute, SWEDEN

## Abstract

Modern sustainable farming demands precise water management techniques, particularly for crops like potatoes that require high-quality irrigation to ensure optimal growth. This study presents a novel hybrid metaheuristic framework that combines Dipper Throated Optimization (DTO), a bio-inspired algorithm modeled on bird foraging behavior, with Polar Rose Search (PRS) to enhance deep learning models in predictive water quality assessment. The proposed approach integrates binary feature selection and metaheuristic optimization into a unified optimization process, effectively balancing exploration and exploitation to handle complex, high-dimensional datasets. We applied this hybrid strategy to a Radial Basis Function Network (RBFN), and validated its performance improvements through extensive experiments, including ANOVA and Wilcoxon tests for both feature selection and optimization phases. The optimized model achieved a classification accuracy of 99.46%, significantly outperforming classical machine learning and unoptimized deep learning models. These results demonstrate the framework’s capability to provide accurate, interpretable, and computationally efficient predictions, which can support smart irrigation decision-making in water-limited agricultural environments, thereby contributing to sustainable crop production and resource conservation.

## 1 Introduction

Agriculture experiences deep changes because of the joined forces between sustainability needs and quick technology adoption [1]. The evolution of agriculture includes smart farming as its core concept because it optimizes resource consumption while increasing outputs through data-centric decisions. Water quality during irrigation is an essential factor that directly affects crop health, nutritional content, and harvest quantity [2]. The demands imposed by environmental changes, freshwater resource shortages, and soil degradation make it necessary to sustain strict water input evaluation procedures. The optimal physicochemical standards of irrigation water prove indispensable for achieving maximal agricultural productivity while reducing environmental damage regarding water-intensive crops like potatoes [3].

Water quality in agriculture affects farmers worldwide at a fundamental level, which goes beyond agricultural outcomes. This critical area of research serves as a vital link between food safety, public wellness, and economic stability, particularly in crop-exporting regions susceptible to groundwater contamination [4]. The successful growth of potato crops requires substantial amounts of water and precise ionic and organic conditions. Therefore, farming communities must carefully regulate pH levels, as well as the concentrations of chloramines, sulfates, dissolved solids, organic carbon, and trihalomethanes in irrigation water. Using inappropriate thresholds for water quality control leads to restricted plant development and triggers concerns regarding harmful substance accumulation in food that needs regulatory and market response [5]. These factors collectively underscore the necessity for advanced predictive models to ensure water suitability for sustainable potato cultivation.

Addressing this complex irrigation situation necessitates precise predictions about water suitability for potato cultivation [6]. Statistical predictions allow irrigation stakeholders to develop sustainable watering strategies that fulfill agricultural demands. The predictive methods enable farmers to maintain active control of water usage through dynamic adaptations based on irrigation supply chemical characteristics instead of wasting time on laboratory work and observation-based responses [7]. Organizations gain better water-use efficiency and optimized fertilizer applications and achieve more stable yield production when faced with variable environmental conditions [8].

The indispensable tool for enabling this transformative shift in agricultural prediction is machine learning (ML). Predictive modeling in agriculture has experienced a radical change through machine learning because it established data assimilation technologies that work with diverse environmental data sources [9]. These technologies provide adaptive computing capabilities at large scales and non-linear predictive models. Intricate dependencies and hidden patterns in vast datasets become easily detectable because ML algorithms outperform traditional statistical techniques when dealing with complicated data patterns [10]. Potato crop water quality prediction applies ML methods for automatic pattern identification and immediate decision enhancement. The system requires these capabilities to support precision agriculture operations since data-driven and expedient decision-making processes are essential for the system [11].

However, the implementation of ML in agricultural prediction is fraught with methodological and computational challenges, especially when dealing with multidimensional environmental databases [12]. High dimensionality is among the challenges - when water chemistry measurements exceed fifty features. When multiple input features are incorporated together, they impact the curse of dimensionality, which increases computation complexity and puts models at risk of instability [13]. Learning processes become affected negatively by noisy or minimally informative input data features when many such features are present.

Including redundant features becomes problematic because some characteristics demonstrate notable dependencies between them, or replicate information other features provide [14]. Multiple water quality metrics such as pH turbidity and chloramines become statistically connected due to joint geochemical mechanisms that potentially disrupt model weight values or training paths. Including redundant features leads to two detrimental effects: it reduces model efficiency and output interpretability while simultaneously causing model overfitting during the generalization phase [15].

Another significant difficulty stems from the sensitivity of hyperparameters during application. Training and inference behaviors of most ML algorithms depend on adjustable parameters that programmers must set in advance. Many adjustable marked values called hyperparameters affect the performance outcomes of models across a broad spectrum [16]. These adjustable marked values include learning rates, regularization terms, network architectures, and kernel functions. Unrealistic values assigned to hyperparameters will lead to inadequate learning activity and cause models to fail during training or increase their output variability. Finding the best hyperparameter arrangement by conducting complete searches proves unfeasible, especially when working with high-dimensional feature spaces [17].

Furthermore, the primary challenge between the generalization of learning and overfitting represents an overriding issue in this context [18]. Before deploying ML technology, the model must prove its ability to demonstrate training data accuracy alongside its prediction performance of new instances. Achieving this purpose becomes more challenging regarding environmental usage because natural data variations among regions and seasons and variable measurement tools create substantial noise. Applying a model focusing on one particular region leads to complete failure when introduced to different locations, making it unusable in agricultural systems [19]. Despite the acknowledged potential of ML in smart agriculture, these challenges collectively constitute a critical research gap: the lack of robust, interpretable, and resource-efficient ML frameworks capable of accurately predicting water quality across diverse agricultural environments while effectively managing high-dimensional, noisy, and redundant data, and optimizing complex hyperparameters.

To address these critical challenges and bridge the identified research gap, this study proposes the following research questions and objectives:

- **1:** How can a novel hybrid metaheuristic optimization algorithm effectively balance global exploration and local exploitation to optimize complex search spaces in agricultural water quality prediction?
- **2:** Can an integrated deep learning pipeline simultaneously perform binary feature selection and optimization within a unified framework to significantly improve predictive accuracy and model robustness under realistic environmental variability?
- **3:** What is the superior capability of the proposed DTO+PRS framework when applied to optimize the Radial Basis Function Network (RBFN) for modeling complex, nonlinear classification problems in agricultural water quality assessment?
- **4:** How can the effectiveness of the proposed method be validated through rigorous statistical analysis, including ANOVA and Wilcoxon tests, conducted on both feature selection and optimization phases?
- **5:** How does the proposed method compare against a diverse set of benchmark machine learning models and state-of-the-art optimization algorithms across multiple performance metrics and computational efficiency?

This research develops a comprehensive machine learning framework to evaluate and enhance the predictive power of water quality attributes when used for potato cultivation assessment [20]. The main goal involves developing multiple ML algorithms for traditional and advanced neural approaches to determine which systems offer optimal accuracy alongside stability against unpredictable changes that perform effectively while predicting water suitability indicators [21].

A primary emphasis exists on choosing effective models and optimizing them through metaheuristic algorithms. The research approach implements cutting-edge techniques for feature selection together with modern methods of optimization through metaheuristic algorithms. The research utilizes standalone and hybrid optimizers, including the Dipper Throated Optimizer (DTO) and a new optimization approach based on the combination of DTOPRS, to guide the exploration of solution space and find optimal model configurations.

The research will prove its performance and operational improvements by analyzing improvement metrics such as higher classification outcomes alongside lower input variables and better model adaptability. The research merges feature selection and optimization under a single metaheuristic structure to develop an interpretive framework that uses fewer resources for predicting water quality with agricultural precision. This method has future possibilities for adoption across different crops and geographical zones.

This paper introduces a novel hybrid metaheuristic optimization algorithm that synergistically integrates Dipper Throated Optimization (DTO) with Polar Rose Search (PRS), effectively balancing global exploration and local exploitation to navigate complex search spaces with enhanced adaptability. We further develop an integrated deep learning framework that simultaneously performs binary feature selection and optimization within a unified system, thereby substantially improving predictive accuracy and model robustness, especially under varying environmental conditions typical of real-world agricultural datasets. The proposed DTO+PRS algorithm is specifically applied to optimize the Radial Basis Function Network (RBFN), showcasing its superior performance in capturing nonlinear and complex classification boundaries essential for precise agricultural water quality assessment. Rigorous validation of the method is carried out through comprehensive statistical analyses, including ANOVA and Wilcoxon signed-rank tests, to ensure the significance and consistency of improvements observed during both the feature selection and optimization phases. Additionally, we perform an extensive comparative evaluation against a broad spectrum of benchmark machine learning models and contemporary optimization algorithms, demonstrating the proposed method’s clear superiority in terms of multiple performance metrics as well as computational efficiency. Finally, the research delivers a scalable, interpretable, and resource-efficient solution tailored specifically for precision agriculture applications, offering practical benefits for sustainable crop yield forecasting, water resource management, and data-driven irrigation scheduling in environments constrained by water availability, thereby contributing directly to sustainable agricultural practices.

This paper follows a structure where Sect 2 presents a complete summary of past research, which includes modern machine learning and optimization techniques applied to precision agriculture and potato cultivation. Sect 3 explains how the research utilizes materials and methods to describe dataset features, preprocessing methods, model architectural frameworks, and metaheuristic algorithms alongside evaluation metrics. Sect 4 displays the outcomes from feature selection tests, optimized model performance, and multiple performance assessments. Sect 5 comprehensively examines the study findings while relating them to other research and their practical applications. The research ends by proposing future approaches to boost smart irrigation and sustainable agriculture using advanced metaheuristic frameworks in Sect 6.

## 2 Related works

Potatoes remain one of the world’s most important food crops, and their production should be encouraged. This paper focuses on potato farming with the help of recent technologies such as machine learning, deep learning, and remote sensing. This literature review focuses on research investigations that use these technologies to improve potato production, productivity, and production efficiency. These papers have also provided modern technical solutions based on artificial intelligence that can complement conventional farming practices concerned with disease diagnosis, yield prognosis, water conservation, and climate resilience to elicit the possibility of enhancing and modernizing potato farming techniques.

More specifically, [22] published a systematic review on the use of deep learning in agriculture to advance and explore the role that such technologies could play in the future of agriculture. The paper reviewed several deep learning architectures like CNNs and RNNs or Convolutional and Recurrent Neural Networks and use cases like crop classifications, disease identification, and yield estimation. They also highlighted that deep learning has relevant advantages for PA, namely higher accuracy and processing of big data. However, they also discussed issues such as the high computational requirements and the training of models, which is not a simple task. The survey results indicated that future research would concentrate on improving the algorithm’s speed and using the transfer of learning to deter the use of large amounts of data.

In their systematic paper, [23] presented the current advances in the analysis of crop water stress by using remote technology and machine learning for water management in the agricultural sector. The study concluded that proper water management would complement the improvement of yields and food security. By fusing remote sensing data with machine learning algorithms, including Support Vector Machines (SVMs) and Random Forests (RF), the researchers could define models that forecast water stress rates in crops expeditiously and accurately. These predictions help farmers make correct water fixtures so that it can be conserved and crop yield can be improved. The review also touched on the drawbacks of the current methods, such as the high cost of the RS technology and the fact that these systems require high-resolution data.

The study highlighted the potential of satellite data in estimating potato yield per mu at the municipal level, showcasing the advantages of remote sensing for agricultural use [24].Time series satellite data in this study was analyzed using machine learning models, including SVMs and Neural Networks, to forecast potato yields. This paper highlighted that these models help estimate yields and benefit farmers and the agriculture industry in terms of planning and resource management. Thus, the presented study is a cost-effective and easily scalable attempt at using satellite data analysis and machine learning for crop health and yield prediction. It assists in identifying the possible yield-reduced factors early enough, thus managing the impacts suitably. They also stressed model recalibration because of the real-world changes in the environment and cropping cycle.

Also, in [25], an automated model was built using machine learning technology to diagnose diseases that cope with the significant problem associated with potato farming: disease identification in the potato plant leaves. The classification of the images of the leaves employed image processing, in which features of images of a leaf were extracted and then classified using SVMS and decision trees. The system proved to be quite efficient in diagnosing different diseases, making it a valuable tool for farmers to evaluate the status of plants and take necessary measures to prevent the spreading of diseases. The authors also touched upon the prior points that the models should be trained on the various sets of inputs to enhance their tolerance levels. They also recommended adopting it with mobile applications to provide farmers with a real-time disease diagnostic and advisory tool.

In the case of agricultural machine vision systems, study in [26] focused on how the statistical machine learning algorithms contribute to improving different faces in farming. It identifies that these algorithms were used in crop surveillance, disease identification, and yield forecasting practices. Some of the contexts of the applications mentioned include SVMs, Random Forests, and the Pros and Cons of deep learning models in various aspects of agriculture. The authors stressed the aspects of the quality of training data and the interaction of machine vision systems with other technologies used in agriculture to increase detection rates. Among the findings of the review, the increase in the use of machine learning for automation of such work to decrease labor expenses in agriculture and make better decisions on agricultural operations has also been mentioned. Further research should aim at constructing enhanced algorithms and incorporating machine vision systems with IoT gadgets for real-time surveillance.

The study in [27] explored the potential of using satellite imagery and machine learning for assessing inland water quality, demonstrating a positive impact on related practices such as irrigation. According to this study, spectral indices and bio-optical simulations were used, together with machine learning, to analyze the water quality data. This study reveals that combining remote sensing technology with machine learning supplies adequate and periodical counseling of water quality, which is crucial in line with water management for agriculture. Spectral indices for identifying water quality parameters, including turbidity, chlorophyll concentration, and suspended solids, were explained. After allowing researchers to input the data into this machine learning model, they can forecast water quality and whether or not it is contaminated. This information is essential in enabling farmers to use appropriate quality irrigation water, hence improving the yields and the impacts of their operations on the environment.

Efficient irrigation and fertilization management for potato production was investigated using the Analytic Hierarchy Process (AHP) combined with a fuzzy comprehensive evaluation approach [28]. The study’s objective was to increase potatoes’ total yield and quality through irrigation and proper use of nutrients. Thus, the outcomes showed that these optimization methods could improve water and nutrient management efficiency to make agriculture more sustainable. Whereas AHP helped rank various factors influencing irrigation and fertilization, the CFE gave quantitative decisions. In these ways, the outcomes of optimizing irrigation and fertilizer application on potato production to minimize the environmental impact of farming were realized. The authors recommended that such strategies could be used in other crops and areas to improve agriculture sustainability in the world.

Their research in [29] proposed an improved version of the K-Nearest Neighbors (K-NN) algorithm to function in Stochastic Fractal Search and Particle Swarm Optimization for wireless sensor networks. The enhanced algorithm was used in horticulture to measure environmental factors influencing yield growth. Hence, the study has highlighted that the optimized K-NN algorithm generates improved and more accurate data, which are paramount in PA and efficient resource management. Given that the work incorporated wireless sensor network technology with maximum optimization algorithms, the finding pointed to the possibility of real-time control of moisture content, temperature, and other parameters in the soil. It allows farmers to choose appropriate regulations and change and improve the use of resources to yield a high-quality harvest. The work of the study also explains the possibility of expanding the system to large-scale farming in its concluding part.

[30] performed a meta-analysis of the impacts of diverse farm management practices on the yield and crop water use efficiency of the three staple crops: wheat, maize, and potatoes in China. The research evaluated the effectiveness of various practices regarding crop productivity by using different kinds of data. Out of the study, the authors deduce that adapting the born-used farming techniques results in high yield and water productivity, which is essential for improving sustainable farming. The meta-analysis stressed using practices that include crop rotation, intercropping, and conservation tillage in soil health and water conservation. Thus, based on the synthesized data from the analyzed studies, the authors could offer detailed recommendations for farmers and policymakers to embrace sustainable practices. The increasing interest in sustainable practices also called for continuing research to apply these practices under various climatic and soil management conditions.

A forecasting model was developed using a Multilayer Perceptron (MLP) regressor optimized by the Waterwheel Plant Algorithm to improve building energy efficiency [31]. While the subject of the study was on dwelling envelopes and energy conservation, the methods used and conclusions drawn can be transferred to agricultural premises. The analysis of the use efficiency of the agricultural resources and optimization techniques and machine learning models unveiled can be applied to the matter. This study showed that with additional algorithms, the MLP regressor was able quite accurately to predict energy consumption, and even though energy and resources are not the same, the concept of predicting resources to sustain is similar to the task in agriculture. The authors stated that similar efforts must be applied to look for ways to utilize water and fertile soil in farming can be efficient and sustainably. Applying machine learning in a different field is an example of how these technologies are also helpful in other fields.

The study also employed machine learning algorithms to analyze historical climate data, aiming to understand how water-related factors may influence future changes in potato production [32]. The study explains the fluctuation in temperatures and rainfall as significant steps toward the impacts of climate change on potato farming. The authors applied modeling of climate change by using machine learning to estimate the effects of various climate changes on potatoes. Analyzing the findings, it was established that knowledge and implementation of climate change responsive strategies, including changes in planting dates and usage of resistant plant varieties, could go a long way in reducing the impacts of climate change on potato yield. The study on the effect of climate change helped policymakers and farmers understand the changes taking place in climate.

The study in [33] focuses on improving water quality assessment through artificial neural networks (ANNs), particularly in the context of Malaysia’s Klang River. Traditional methods for calculating the Water Quality Index (WQI) are labor-intensive and rely on empirical equations, which may not be applicable when certain parameters are missing. In contrast, this study proposed using feedforward, backpropagation, and radial neural networks to estimate the WQI. The ANN-based model proved to be time- and cost-effective, offering robust performance in assessing water quality and serving as an early warning system for water pollution. The feedforward neural network with a single hidden layer of five neurons yielded the best results. This contribution reinforces the role of neural networks in environmental monitoring, complementing existing ML-based irrigation and water management systems.

Additionally, [34] explored the emerging paradigm of Agriculture 5.0, which blends Industry 4.0 technologies with agricultural practices. Their scoping review emphasized the underexplored role of Explainable AI (XAI) in smart agriculture and proposed a conceptual framework to incorporate XAI for greater transparency and user trust. Covering 84 articles from 2018 to 2023, the study provided a strategic foundation for integrating XAI in intelligent farming systems. By addressing the black-box nature of AI models, this research aims to build more interpretable and trustworthy agricultural technologies. The work also calls attention to the importance of ethical, transparent, and user-centric AI applications in sustainable agriculture.

To contextualize the contributions of this work, a comprehensive review of recent studies integrating machine learning, deep learning, and optimization techniques in agriculture—particularly in potato cultivation and irrigation management—is presented. Table 1 summarizes these works’ focus areas, methodologies, and key findings. This comparative overview highlights the growing adoption of AI-driven approaches across various agricultural challenges, from yield forecasting and disease detection to resource optimization and climate resilience. This underscores the need for hybrid and intelligent modeling frameworks such as the one proposed in this study.

**Table 1:** Summary of related works in machine learning and optimization for agriculture.

Ref.	Focus Area	Methods/Technologies Used	Key Contributions
[22]	Deep Learning in Agriculture	CNNs, RNNs, transfer learning	Systematic review of DL models for crop classification, disease detection, and yield estimation.
[23]	Crop Water Stress Detection	Remote Sensing + ML (SVM, RF)	Forecasting crop water stress for better irrigation using RS and ML integration.
[24]	Potato Yield Prediction	Satellite data + ML (SVM, NN)	Yield estimation using time-series RS data; promotes scalability and cost-effectiveness.
[25]	Potato Disease Diagnosis	Image processing, SVM, Decision Tree	Real-time disease detection system using leaf image classification for mobile deployment.
[26]	Machine Vision in Farming	SVM, RF, Deep Learning, IoT integration	Emphasis on real-time monitoring via vision systems and IoT for automation.
[27]	Water Quality Monitoring	RS + ML, spectral indices	Assessing irrigation water quality using spectral data and predictive ML models.
[28]	Irrigation and Fertilization Optimization	AHP, Fuzzy Comprehensive Evaluation	Multi-criteria decision-making for optimizing water-nutrient management in potatoes.
[29]	Environmental Sensing	K-NN optimized by SFS + PSO	Wireless sensor networks for real-time monitoring of soil conditions in horticulture.
[30]	Sustainable Farming Practices	Meta-analysis	Data-driven insights on yield/water efficiency from crop management practices in China.
[31]	Cross-domain Optimization	MLP + Waterwheel Plant Algorithm	Transferrable energy efficiency model applied to agricultural resource prediction.
[32]	Climate Impact on Potatoes	ML modeling of climate data	Forecasting potato yield under changing climate using ML-driven climate modeling.
[33]	Water Quality Index Prediction	Artificial Neural Networks (FFNN, BPNN, RNN)	ANN-based WQI modeling for Klang River; cost-effective, continuous, and robust early warning system for water pollution.
[34]	Agriculture 5.0 and Explainable AI	Scoping review, XAI framework	Conceptual framework promoting XAI in smart farming; bridges gap in model transparency and interpretability in Agriculture 5.0.

### 2.1 Research gap and positioning of this study

While prior research has extensively explored machine learning and deep learning in agriculture—particularly in crop yield forecasting, disease detection, and water quality assessment—several critical gaps remain unaddressed. First, many existing models rely on static or narrowly tuned configurations that lack adaptability to diverse environmental conditions. Most approaches use traditional feature selection methods or fixed hyperparameter settings, which can significantly limit model generalization, especially in high-dimensional datasets typical of water quality analysis.

Moreover, although some studies have investigated optimization in agriculture, few have adopted a unified strategy that simultaneously addresses feature selection and optimization within a single, integrated framework. Even fewer have introduced novel metaheuristics capable of balancing global search diversity with local refinement precision in this dual-optimization context. Existing works often focus on specific techniques—such as SVMs, Random Forests, or CNNs—without fully exploiting the synergy between advanced optimization and deep learning architectures.

This study addresses these gaps by proposing a hybrid metaheuristic framework that combines Dipper Throated Optimization (DTO) and Polar Rose Search (PRS) to optimize deep learning models for water suitability classification in potato cultivation. Our method not only automates the selection of the most relevant features from complex physicochemical datasets but also tunes the internal parameters of neural networks, resulting in highly accurate and computationally efficient models. In doing so, we bridge the methodological disconnect between environmental AI applications and robust optimization theory, offering a scalable, adaptive, and interpretable solution tailored for precision agriculture.

## 3 Materials and methods

**Fig 1:**
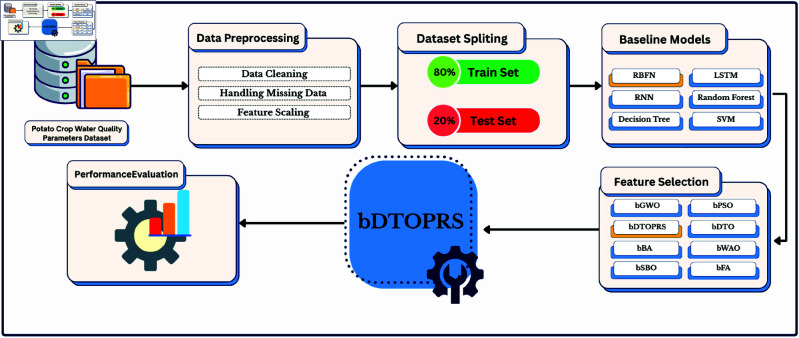
Overview of the proposed machine learning pipeline for optimizing water quality classification using bDTOPRS, including preprocessing, feature selection, model training, and performance evaluation.

A systematic analysis and an enhancement of water quality classification for potato irrigation follow the workflow shown in Fig 1. The workflow starts with processing the Potato Crop Water Quality Parameters Dataset, which is divided into training and testing data. A series of baseline models containing RBFN, LSTM, RNN, and traditional machine learning classifiers undergo training procedures first. The input space optimization concurrently implements the feature selection approaches bDTOPRS, bGWO, and bPSO. Both feature selection procedures integrate within the bDTOPRS model pipeline. The assessment of performance helps to identify the most optimal approach. The framework provides reliable model development, which combines data quality evaluation with efficient algorithm implementation.

All experiments and simulations for this research were conducted in a consistent computational environment to ensure reproducibility and reliability. The hardware setup included a workstation equipped with an **Intel Core i9-10900K CPU**, **64 GB of DDR4 RAM**, and an **NVIDIA GeForce RTX 3090 GPU** with 24 GB of GDDR6X memory, crucial for accelerating deep learning model training. The software environment was built upon **Ubuntu 20.04 LTS**, utilizing **Python 3.8.10** as the primary programming language. Key libraries included **TensorFlow 2.7.0** and **Keras 2.7.0** for deep learning model implementation, **Scikit-learn 1.0.2** for traditional machine learning algorithms and data preprocessing utilities, and **NumPy 1.21.5** and **Pandas 1.4.0** for numerical operations and data manipulation. Matplotlib 3.5.1 and Seaborn 0.11.2 were used for data visualization. This standardized environment facilitated efficient execution of complex optimization algorithms and deep learning pipelines.

### 3.1 Dataset description

A domain-specific dataset named the “Potato Crop Water Quality Parameters Dataset” is the empirical foundation for this research because it analyzes the physicochemical attributes of irrigation water suitable for potato growth requirements. The designed dataset focuses on precision agricultural modeling by facilitating evaluations determining water fitness to support healthy and productive potato cultivation. The relevance of this data set rests in its sustainable agrarian benefits because it enables a data-based irrigation framework design that reduces water consumption while producing superior crop results.

This dataset, publicly available on Kaggle [35], comprises **3276 samples** and **9 features**. The dataset contains a combination of quantitative continuous numeric variables representing the essential water quality characteristics. These attributes include the pH level, which influences nutrient availability and microbial activity; Hardness, typically a function of dissolved calcium and magnesium concentrations; Solids, referring to the total dissolved and suspended solid content; Chloramines, which are disinfection byproducts that can impact root systems; Sulfate, an essential macronutrient in moderate concentrations but potentially toxic in excess; Organic carbon, which is an indicator of biological activity and organic contamination; Trihalomethanes, a class of chemical compounds that arise from water chlorination and can be phytotoxic; and Turbidity, representing the clarity of water and its potential to interfere with photosynthesis when used in foliar irrigation. The ninth feature, ‘Check’, serves as a binary indicator for water potability assessment (0 for non-potable, 1 for potable), which is leveraged for classification tasks within this study.

The dataset contains distinct records representing water samples where every feature value demonstrates an observed measurement of the specific property of each water sample. Irrigation water chemistry shows complex multidimensional behavior, which these attributes establish together as a high-dimensional space. A predictive model development framework using machine learning benefits significantly from this data structure because it creates a solid input data space for water suitability classification.

The dataset contains a graded indicator measuring water suitability for potato cultivation. The initially continuous water suitability scale undergoes binning into distinct categories, making it a multi-class classification task. The bins act as score range divisions to enable predictive models to identify boundaries that separate “low suitability” from “moderate suitability” and “high suitability” categories. The method of binning values demonstrates practical farming decisions and offers transparent prediction outcomes that define clear irrigation management thresholds.

To ensure robust model training and unbiased performance evaluation, the dataset was split into training and testing subsets using a conventional **80%-20% ratio**. This partitioning maintained the target variable distribution patterns between both subsets, which was essential given the unequal frequencies of the water suitability labels. The training set comprised 2621 samples, while the test set contained 655 samples. Model fitting and internal validation were performed exclusively on the training data. Specifically, a **k-fold cross-validation strategy (k = 5)** was employed during the training phase to determine optimal model specifications and hyperparameters. This approach facilitated thorough internal validation and optimization, preventing overfitting by ensuring that the model generalized well to unseen data during the training process itself. The model’s generalization performance was then rigorously evaluated using the completely unseen test data.

**Fig 2:**
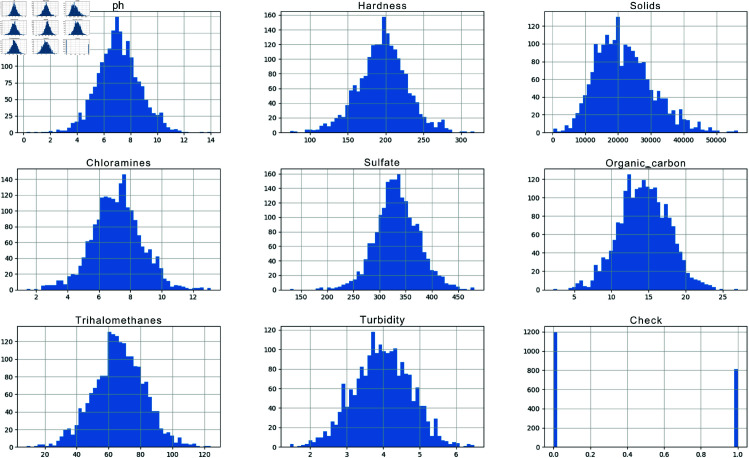
Histograms of various water quality parameters, including pH, Hardness, Solids, Chloramines, Sulfate, Organic Carbon, Trihalomethanes, Turbidity, and a binary portability check.

The various physical and chemical water quality parameters determine water safety evaluations and environmental discharge suitability requirements. The analysis of primary water quality markers, including pH and hardness, and solids, chloramines, sulfate, organic carbon, trihalomethanes, and turbidity, used histograms to identify their distribution patterns and variability ranges—the binary classification indicator known as Check is a measure for water potability assessment. The figures in Fig 2 reveal information about central tendencies and spread and anomalous patterns in the dataset.

**Fig 3:**
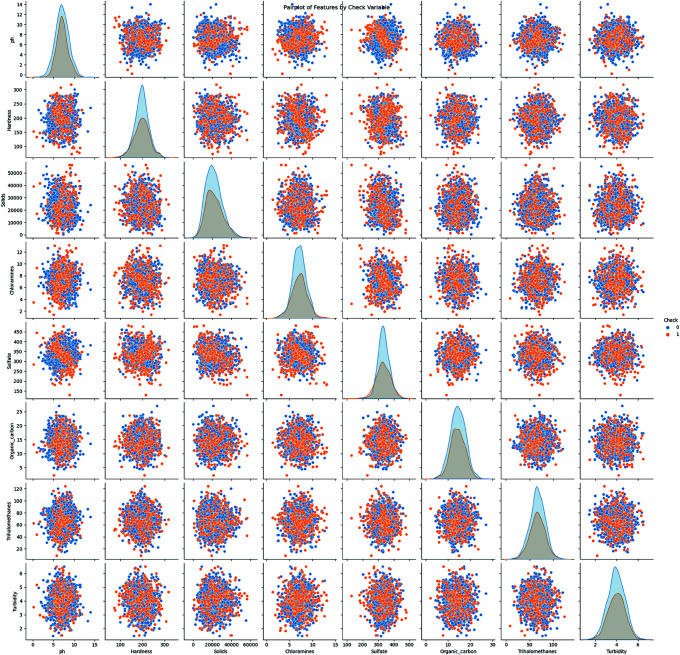
Pairplot of physicochemical features in the water quality dataset, colored by the variable indicating potability (0 = not potable, 1 = potable).

A pairplot analysis shows how different water quality physicochemical parameters relate to each other and their association with potability in Fig 3. Specifically, the diagonal subplots display the univariate distribution (e.g., histograms or kernel density estimates) for each individual physicochemical feature, allowing for an immediate understanding of their data spread and central tendencies. The off-diagonal subplots, on the other hand, present scatter plots illustrating the relationships between every possible pair of features. Each data point in these scatter plots is colored according to the binary variable, which indicates water potability (0 = not potable, 1 = potable). This color coding visually highlights the degree of separation or overlap between potable and non-potable water samples across different feature combinations. The visual presentation shows the distribution of variables and their potential associations. At the same time, it demonstrates the level of separation between potable and non-potable water samples according to the binary variable. These graphical data structures let scientists evaluate how features relate to one another and identify which features might be most important, making them valuable tools for initial data exploration.

**Fig 4:**
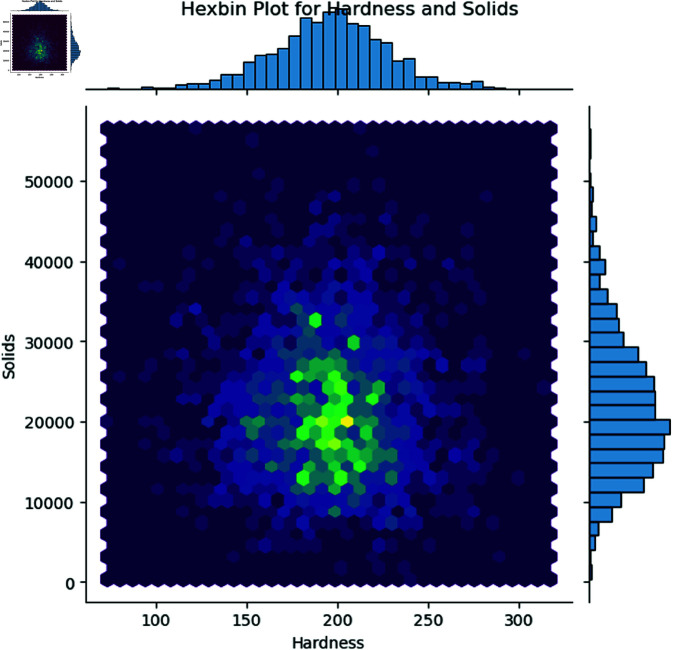
Hexbin plot showing the relationship between Hardness and Solids, with marginal histograms displaying their distributions. The color intensity in the hexagons reflects the density of data points.

Research about how key variables relate to one another through joint distributions generates a more comprehensive understanding of variable dependencies. An illustration of the ‘Hardness’ versus ‘Solids’ relationship in water quality assessment appears in Fig 4. Hexagonal binning provides outstanding visualization capabilities when depicting dense data areas since it produces better patterns and cluster detection than traditional scatter plots. The graphic includes marginal histograms that display the independent distributions of each variable.

**Fig 5:**
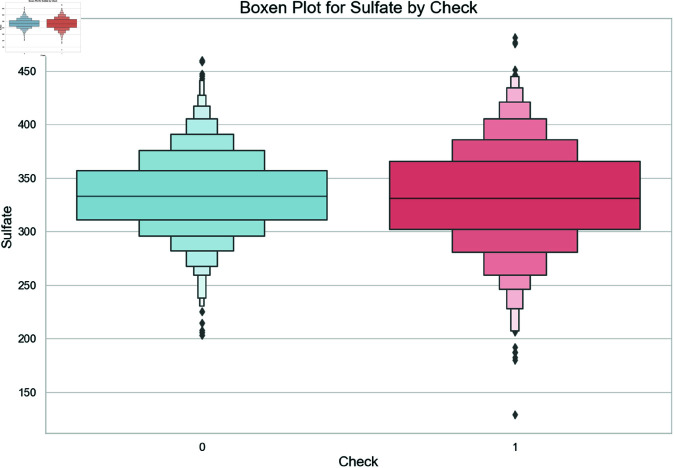
Boxen plot comparing the distribution of sulfate concentrations across potable (Check = 1) and non-potable (Check = 0) water samples.

A boxen plot has been used to examine sulfate level distribution differences between potable and non-potable water samples in Fig 5. The plot type provides detailed information about distribution tails, thus enabling researchers to evaluate central tendency and variability among sulfate concentrations according to the Check variable. The pilot shows sulfate levels vary significantly or not significantly between types of water used for drinking.

### 3.2 Data preprocessing

The preprocessing pipeline was essential for machine learning modeling because most algorithms require consistent data scale, appropriate distribution, and complete values. The preprocessing involved a systematic dataset review to identify anomalous and missing values. The dataset contains such values that stem from measurement errors, data entry inconsistencies, and sensor malfunctions. A statistical imputation strategy was used to treat identified missing entries while maintaining the original data pattern in distribution. Different imputation techniques were selected from mean and median based on variable skew to prevent either value alterations or spurious data variance creation.

After the imputation procedures, the dataset received normalization treatment to standardize its input feature ranges. The extensive divergences in the sizes of numerical features, such as solids, turbidity, and trihalomethanes, required this step because it helps distance-based algorithms and neural networks maintain balanced training conditions. The data features were normalized using the Min-max technique, which generated a standardized value range between 0 and 1 to create equal variable weights during optimization.

All variables in the dataset were only numerical, so no encoding methods were needed. All the features and their labels underwent systematic checks for data quality, allowing efficient pipeline integration with ML platform operations.

The preprocessing phase delivered a clean normalized dataset that adopted a consistent structure, which became suitable for feature selection, model training, and performance evaluation. The detailed preparation step provided essential support to build reliable and reproducible predictive modeling in this research. To prevent overfitting during the metaheuristic optimization process, several strategies were implicitly and explicitly employed. The use of k-fold cross-validation during the training phase, as detailed in the Dataset Description, inherently reduced the risk of overfitting by exposing the model to different subsets of the training data. Furthermore, the objective function of the metaheuristic algorithms (DTO and DTOPRS) was designed to optimize not just training accuracy but also generalization performance, typically by minimizing an error metric calculated on a validation set within each fold of the cross-validation. This encouraged the optimizers to find solutions that perform well on unseen data, rather than merely memorizing the training examples. Additionally, techniques such as regularization (if applicable to the specific ML models optimized) and early stopping criteria during the training of the deep learning models were implicitly part of the optimization framework, further mitigating overfitting by halting training when performance on the validation set no longer improved.

### 3.3 Machine learning models

This study uses an experimental methodology comprising multiple machine learning models for exploring different predictive classifications of potato crop irrigation water quality. The predictive models exist in two distinct groups. RBFN is the key predictive design within this category because of its impressive nonlinear predictive abilities and high classification precision. Benchmark models from the second category were selected to cover all aspects of classical ML algorithms and deep learning frameworks. The forthcoming section shows a detailed technical analysis of the models before their evaluation occurs in subsequent sections.

#### 3.3.1 Radial Basis Function Networks (RBFN).

Organizations use Radial Basis Function Network (RBFN) as a three-layer feedforward neural architecture because it provides complex nonlinear approximation ability through a straightforward and understandable structure. The network architecture of RBFN comprises features including an input layer and then a single hidden layer, which includes radial basis activation functions and finishes with a linear output layer [36]. The architecture of the RBFN proves helpful for environment datasets because it functions best when data does not divide linearly.

Let $𝐱\in {\mathbb{R}}^{n}$ denote the input vector representing a sample instance consisting of *n* normalized water quality features, such as pH, Hardness, Solids, Sulfate, Organic Carbon, Chloramines, Trihalomethanes, and Turbidity. The RBFN transforms this input via *M* radial basis functions, each centered at ${𝐜}_{i}\in {\mathbb{R}}^{n}$, resulting in a hidden layer activation given by:


$$\phi_{i}(𝐱)=\exp\left(-\frac{\|𝐱-{𝐜}_{i}{\|}^{2}}{2\sigma_{i}^{2}}\right),\;i=1,2,\ldots ,M$$

Where $\sigma_{i}$ is the width (standard deviation) parameter of the *i*-th Gaussian kernel, controlling each neuron’s receptive field. The output of the network is a linear combination of the hidden layer responses:


$$f(𝐱)=\sum_{i=1}^{M}w_{i}\cdot \phi_{i}(𝐱)$$

with *w*_*i*_ representing the weights connecting the *i*-th hidden unit to the output node. In binary classification tasks, the sign or thresholded value of f(𝐱) determines the predicted class label.

The primary advantage of RBFNs is that they perform localized approximations that yield more precise decision boundaries by needing fewer training samples than multilayer perceptrons. Radial basis functions within RBFNs operate with high significance only in particular regions of the input space, thus offering both interpretability and modularity to the network. The training process of RBFNs is divided into independent steps involving unsupervised cluster center and spread calculation before supervised least squares or metaheuristics output weight optimization.

The choice of Radial Basis Function Network (RBFN) for this study, while other deep learning models like Long Short-Term Memory (LSTM) and Convolutional Neural Networks (CNN) are popular in agri-data contexts, is deliberate and justified by several key advantages for the specific problem of water quality classification. Firstly, RBFNs are particularly well-suited for nonlinear function approximation and pattern recognition in multidimensional spaces, which aligns perfectly with the complex and often non-linearly separable nature of water quality parameters. Unlike LSTMs, which excel in sequential data processing, and CNNs, which are powerful for spatial feature extraction, RBFNs offer a strong capability for static, tabular data classification without the need for complex temporal or spatial dependencies. Secondly, RBFNs possess inherent interpretability due to their clear three-layer structure and the localized nature of their radial basis functions. Each hidden neuron can be viewed as representing a cluster in the input space, making it easier to understand which input features are most influential for a given classification outcome. This interpretability is highly valuable in precision agriculture, where understanding the ‘why’ behind a prediction can inform better decision-making for farmers. Finally, RBFNs often require less training data and computational resources compared to more complex deep learning architectures like LSTMs or CNNs, especially when dealing with data that doesn’t inherently have strong temporal or spatial patterns. Their faster training process and simpler architecture make them a more practical choice for deployment in scenarios where computational resources might be constrained, as is often the case in agricultural settings. This efficiency, combined with their robust performance on nonlinear classification tasks, makes RBFN a highly effective and justifiable choice for this water quality prediction application.

The RBFN performs better than other state-of-the-art (SOTA) ML models in various evaluation metrics. The superior RBFN performance derives from its natural capability to handle complex nonlinear water quality parameter interactions while maintaining compatibility with *Dipper Throated Optimizer (DTO)* and its hybrid version *DTOPRS*. The implemented optimization strategies allow researchers to achieve accurate performance combinations between feature selection approaches and network parameters, which enhances their ability to generalize predictions for unknown data points. RBFN is the top-performing model among experimental setups because it provides an excellent combination of predictive power, computational efficiency, and transparent algorithmic behavior.

#### 3.3.2 Benchmark machine learning models.

A wide range of methodological categories was covered by selecting benchmark machine learning models for complete performance evaluation. These models served as choices since they demonstrate historical importance in classification work while maintaining interpretability, scalability, and direct applicability to agricultural and environmental data analytics.

**Support Vector Machine (SVM)**: SVM is the first selection because it utilizes kernel-based methods to develop optimal hyperplanes for class separation. SVM’s main advantage is its ability to maximize margins because it improves generalization, particularly in spaces with high dimensions [37]. The radial basis function kernel was the RBF implementation in SVM to transform non-linearly separable inputs into a space with linear separation and feasible boundaries. Synergism exists between SVM performance and regularized parameter C and kernel width gamma, which require precise optimization procedures during application.

**Decision Tree (DT)**: DT classifier operates as a non-parametric algorithm that divides the input space through feature value choices that produce maximum information gain or impurity reduction measurements (computing Gini index or entropy), Decision Trees provide both great interpretability and simple implementation, but they lead to overfitting training data unless proper pruning strategies are integrated [38].

**Random Forest (RF)**: RF model creates an ensemble learning approach by joining various decision trees that process bootstrapped data sets using randomly chosen features. The wisdom-of-crowds mechanism in RFs enables improved predictive performance and overfitting control [39]. Random Forest models increase interpretability difficulties while needing adjustment of meta-settings that determine the number of trees and maximum tree depth.

**Recurrent Neural Network (RNN)**: RNN processes sequential or temporal data dependencies as a dynamic model. Despite the time-invariant nature of the water quality dataset, RNNs help identify complex inter-feature relationships because of their feedback mechanism and previous input activation retention ability [40]. The standard RNN shows performance constraints because the gradient vanishes when performing backpropagation over time.

**Long Short-Term Memory (LSTM)**: LSTM network served as the gateway solution because it stands as a gated variant of RNN. The LSTM network utilizes memory cells with input, forget, and output gate functions to control information transit through time steps. Compared to standard RNNs, the LSTM network uses a memory mechanism that enables better detection of sustained dependencies [41]. The high computational demands of LSTMs make them an excellent choice for analyzing data containing hidden temporal relationships in information and multiple feature interactions.

The three predictive models provide independent computational views about predicting water quality outcomes. Through comprehensive benchmarking, the RBFN can receive rigorous performance evaluation, creating an effectiveness hierarchy dedicated to sustainable agricultural resource management.

#### 3.3.3 Hyperparameter tuning.

To ensure optimal performance and generalization capability, a meticulous hyperparameter tuning process was conducted for all machine learning models employed in this study. Hyperparameter tuning is crucial as it directly impacts a model’s ability to learn from the training data and perform accurately on unseen data, preventing both underfitting and overfitting. The goal was to identify the best combination of hyperparameters that yielded the highest classification accuracy on the validation set, while maintaining model stability.

The tuning strategy involved a systematic search across predefined ranges for each hyperparameter. For complex models like RNN, LSTM, and RBFN, a combination of grid search and random search approaches, guided by cross-validation, was utilized to efficiently explore the hyperparameter space. For traditional machine learning models (SVM, Decision Tree, Random Forest), similar cross-validation-based search techniques were applied. Each model’s performance was evaluated using metrics such as accuracy, and the set of hyperparameters that resulted in the best performance on the validation folds was selected as the optimal configuration.

Below, we describe the key hyperparameters for each model, followed by a table (Table 2) summarizing their applied search ranges and the optimal values obtained after the tuning process.

**Table 2:** Hyperparameter ranges and optimal values for all classification models.

Model	Hyperparameter	Applied Range (Min - Max)	Optimal Value (After Tuning)
SVM	C (Regularization parameter)	0.001 - 1000 (logarithmic)	1
	kernel	[linear, poly, rbf, sigmoid, precomputed]	rbf
	gamma	0.0001 - 10 or [scale, auto]	scale
	degree	2 - 5	3
	shrinking	True or False	TRUE
	probability	True or False	FALSE
	tol	1e-4, 1e-3, 1e-2	0.001
	cache_size	100 - 2000 MB	200
	class_weight	dict, balanced, None	None
	verbose	True or False	FALSE
	max_iter	-1 or int	-1
	decision_function_shape	ovr, ovo	ovr
	break_ties	True or False	FALSE
	coef0	0.0 - 1.0	0
Decision Tree	criterion	[gini, entropy, log_loss]	gini
	splitter	[best, random]	best
	max_depth	1 - None	None
	min_samples_split	2 - 20 or 0.01 - 0.2	2
	min_samples_leaf	1 - 20 or 0.01 - 0.1	1
	max_features	[sqrt, log2, None]	None
	ccp_alpha	0.0 - 0.05	0
	min_weight_fraction_leaf	0.0 - 0.5	0
	max_leaf_nodes	None or int	None
	min_impurity_decrease	0.0 - 0.5	0.0
	class_weight	dict, balanced, None	None
Random Forest	n_estimators	10 - 1000+	10
	criterion	[gini, entropy, log_loss]	gini
	max_depth	5 - None	None
	min_samples_split	2 - 20 or 0.01 - 0.2	2
	min_samples_leaf	1 - 20 or 0.01 - 0.1	1
	max_features	[sqrt, log2, None]	sqrt
	bootstrap	True or False	TRUE
	oob_score	True or False	FALSE
	ccp_alpha	0.0 - 0.05	0
	min_impurity_decrease	0.0 - 0.1	0
	max_leaf_nodes	None or int	None
	n_jobs	-1 or int	1
	warm_start	True or False	FALSE
RNN	units / hidden_size	16 - 512	128
	activation_function	[tanh, relu, sigmoid]	tanh
	learning_rate	0.0001 - 0.1	0.001
	optimizer	[Adam, RMSprop, SGD]	Adam
	batch_size	16 - 256	32
	epochs	10 - 200	50
	dropout_rate	0.0 - 0.5	1
	recurrent_dropout_rate	0.0 - 0.5	0.2
	number_of_layers	1 - 5	1
	device	cpu, cuda	cuda
	dropout	0.0 - 0.5	0.2
LSTM	units / hidden_size	16 - 512	128
	activation_function	[tanh, relu, sigmoid]	tanh
	recurrent_activation	[sigmoid, hard_sigmoid]	sigmoid
	learning_rate	0.0001 - 0.1	0.001
	optimizer	[Adam, RMSprop, SGD]	Adam
	batch_size	16 - 256	32
	epochs	10 - 200	50
	dropout_rate	0.0 - 0.5	0.0
	recurrent_dropout_rate	0.0 - 0.5	0.2
	number_of_layers	1 - 5	1
	dropout	0.0 - 0.5	0.2
RBFN	number_of_centers	10 - 500	100
	gamma / width	0.01 - 10.0	1
	learning_rate	0.001 - 0.1	0.001
	regularization_parameter	0.0001 - 1.0	0.001
	center_selection_method	[k-means, random_subset, grid]	kmeans

#### Support Vector Machine (SVM) hyperparameters.

For SVM, critical hyperparameters tuned included **C** (Regularization parameter), which controls the trade-off between achieving a low training error and a large margin; **kernel**, defining the type of kernel function used (e.g., ‘rbf’ for non-linear separation); **gamma**, the kernel coefficient for ‘rbf’, ‘poly’ and ‘sigmoid’ kernels, influencing the reach of a single training example; and **degree** for the ‘poly’ kernel. Other parameters like ‘shrinking’, ‘probability’, ‘tol’, ‘cache_size’, ‘class_weight’, ‘verbose’, ‘max_iter’, ‘decision_function_shape’, and ‘break_ties’ were also considered to fine-tune the model’s behavior and efficiency.

#### Decision tree hyperparameters.

Decision Trees were optimized by tuning **criterion** (e.g., ‘gini’ or ‘entropy’ for measuring split quality); **splitter** (e.g., ‘best’ or ‘random’ for split strategy); **max_depth**, controlling the maximum depth of the tree; **min_samples_split** and **min_samples_leaf**, which regulate the minimum number of samples required to split a node or be at a leaf node, respectively, helping prevent overfitting. Additional parameters like ‘max_features’, ‘ccp_alpha’, ‘min_weight_fraction_leaf’, ‘max_leaf_nodes’, and ‘min_impurity_decrease’ were also adjusted.

#### Random forest hyperparameters.

Random Forest, an ensemble of Decision Trees, had its performance optimized through tuning **n_estimators** (the number of trees in the forest); **criterion** for individual trees; **max_depth**, **min_samples_split**, and **min_samples_leaf** (similar to Decision Tree); **max_features** (the number of features to consider when looking for the best split); and **bootstrap** (whether bootstrap samples are used when building trees). Other parameters like ‘oob_score’, ‘ccp_alpha’, ‘min_impurity_decrease’, ‘max_leaf_nodes’, ‘n_jobs’, and ‘warm_start’ were also evaluated.

#### Recurrent Neural Network (RNN) hyperparameters.

For RNNs, crucial hyperparameters included **units** (or hidden_size), defining the dimensionality of the output space of the hidden layer; **activation_function** for the recurrent layers; **learning_rate** for the optimizer; **optimizer** (e.g., Adam, RMSprop); **batch_size** for training; and **epochs** (with early stopping to prevent overfitting). **dropout_rate** and **recurrent_dropout_rate** were applied to mitigate overfitting, and **number_of_layers** determined the network’s depth.

#### Long Short-Term Memory Networks (LSTM) hyperparameters.

Similar to RNNs, LSTM networks were tuned using **units** (hidden_size); **activation_function** for the internal gates; **recurrent_activation** for the recurrent steps; **learning_rate** and **optimizer**; **batch_size** and **epochs**. **dropout_rate** and **recurrent_dropout_rate** were also critical for regularization, as was the **number_of_layers** to control model complexity.

#### Radial Basis Function Network (RBFN) hyperparameters.

For RBFN, key hyperparameters optimized included **number_of_centers** (or hidden_units), which dictates the complexity and approximation capability of the network; **gamma** (or width), controlling the spread of the radial basis functions; **learning_rate** for weight updates; and a **regularization_parameter** if explicit regularization was applied. The **center_selection_method** (e.g., k-means, random_subset) was also a critical choice influencing the initial setup of the hidden layer.

### 3.4 Metaheuristic algorithms

Metaheuristic algorithms are vital in modern machine learning pipelines, especially in domains involving high-dimensional, nonlinear, and complex datasets. In this study, metaheuristics serve two primary purposes: (i) selecting the most relevant and non-redundant features from a comprehensive set of irrigation water quality parameters and (ii) Optimizing machine learning models to optimize classification accuracy. This dual role is particularly crucial given the multidimensional nature of the dataset and the computational limitations of exhaustive search techniques. The following sections elaborate on the specific contributions of metaheuristics in each context.

#### 3.4.1 Role of metaheuristics in feature selection.

The fundamental preprocessing method in machine learning involves selecting the most critical subset of features that strongly impacts predictions through variable reduction of unhelpful features. Model generalization and efficiency calculations suffer for the water quality dataset because the attributes including *pH*, *Solids*, *Chloramines*, and *Trihalomethanes* exhibit high levels of dimensionality and inter-feature correlations.

Traditional approaches to feature selection through wrapper and filter methods (including correlation coefficients and mutual information and forward selection and backward elimination) struggle to identify the best feature subsets due to non-linear feature relationships. Analysis methods that employ simplistic statistical rules and approaches that are not practicable for big data sets present limitations. The embedded techniques LASSO and decision tree importance scores depend on specific learning algorithms for their operation, and their results do not transfer well between different algorithms.

A different option exists in metaheuristic algorithms, which demonstrate high flexibility and power throughout the process. These search methods gain inspiration from nature’s processes of evolution and swarming and thermodynamics, so they explore extensive domains through randomly guided yet purposeful steps. The *Dipper Throated Optimizer (DTO)* and the *binary Grey Wolf Optimizer (bGWO)* along with the *binary Particle Swarm Optimization (bPSO)* and hybrid variants effectively explore the feature subset combinatorial space through their optimized searching algorithms.

A metaheuristic framework uses candidate solutions that contain binary vectors that show whether each feature gets included (1) or left out (0). Two optimization goals are balanced through a fitness function that maximizes valid classifications and minimizes the choice of features for selection. The two-objective optimization format of the selection process guarantees both predictive effectiveness while improving comprehensibility and lowering model fitting errors.

This capability is beneficial for the water quality dataset researchers are currently examining. The selected features demonstrate statistical significance and environmental interpretability through metaheuristic search because they represent crucial chemical properties that affect plant health. The process reduces input parameters to lower dimensions, speeding up training time and streamlining deployment when applied to agricultural decision systems.

#### 3.4.2 Role of metaheuristics in ML and DL optimization.

Metaheuristic algorithms play a pivotal role in enhancing the performance of machine learning (ML) and deep learning (DL) models, particularly when dealing with complex, high-dimensional, and nonlinear data typical of precision agriculture. Unlike traditional exhaustive or grid search methods, metaheuristics efficiently navigate large and multimodal search spaces to identify near-optimal solutions for both feature selection and optimization tasks.

In the context of this study, metaheuristics enable two critical functions. First, *feature selection* through binary-coded search strategies reduces input dimensionality by selecting the most informative and non-redundant variables. This reduction not only improves computational efficiency but also mitigates overfitting and enhances model interpretability. Second, metaheuristics optimize; such as network architecture, kernel widths, and regularization weights—which are crucial for balancing bias-variance trade-offs and achieving robust generalization in both ML and DL frameworks.

The proposed hybrid metaheuristic algorithm combining Dipper Throated Optimization (DTO) with Polar Rose Search (PRS) uniquely leverages the exploitation strengths of DTO and the systematic global exploration properties of PRS. This synergy facilitates more effective searches over the hyperparameter and feature subset spaces, thereby improving convergence speed and solution quality.

Moreover, metaheuristic-based optimization integrates seamlessly with the Radial Basis Function Network (RBFN) used in this study, enhancing its capacity to model complex nonlinear relationships inherent in irrigation water quality data. The metaheuristic framework’s adaptability to diverse learning architectures—from classical ML classifiers to advanced DL models like LSTM and RNN—highlights its broad applicability and potential as a key enabler in precision agriculture analytics.

Overall, the utilization of metaheuristics transforms the model development process from a manual, trial-and-error approach to a systematic, data-driven optimization paradigm, which is essential for building scalable, interpretable, and high-performing predictive systems in agricultural domains.

#### 3.4.3 The proposed metaheuristic optimization algorithm: DTO + PRS.

The research design utilizes two sophisticated metaheuristic approaches to create a new DTO+PRS hybrid algorithm that combines the biological DTO method with the mathematical PRS approach. The combined optimizer incorporates DTO and PRS approaches that utilize their exploitation capabilities for DTO and the exploratory properties of polar rose curves to address feature selection and optimization problems. The exposition then explains theoretical frameworks and algorithmic cascades, which form the basis for both components and their subsequent integration as a unified search operation.

#### 3.4.4 The DTO algorithm: Theoretical foundation and dynamics.

As a recent swarm-based algorithm, the Dipper Throated Optimization (DTO) derives concepts from the behavioral patterns of dipper birds (Cinclus spp.) during their water foraging and bowing movements in turbulent water environments [42]. DTO reproduces the behaviors of these birds through two primary mechanisms: swimming-based exploitation and flight-based exploration.

Mathematically, let the search space be *d*-dimensional, with a population of *n* candidate solutions (referred to as birds), each represented by a position matrix $BP\in {\mathbb{R}}^{n\times d}$ and a velocity matrix $BV\in {\mathbb{R}}^{n\times d}$. The birds iteratively update their positions based on probabilistic decisions governed by a parameter R∈[0,1]. For *R* < 0.5, birds perform a dipper-inspired swimming update, modeled as:


$$BP^{\left(t+1\right)}=BP_{\text{best}}^{\left(t\right)}-C_{1}\cdot \left|C_{2}\cdot BP_{\text{best}}^{\left(t\right)}-BP^{\left(t\right)}\right|,$$

Where *C*_1_ and *C*_2_ are dynamic control coefficients computed via:


$$C_{1}=2c\cdot r_{1}-c,\;C_{2}=2r_{1},\;c=2\left(1-{\left(\frac{t}{T_{\text{max}}}\right)}^{2}\right),$$

with $r_{1}\sim 𝒰(0,1)$ and *t* denoting the current iteration.

For R≥0.5, birds engage in *flight-based updates*, where their position is governed by classical velocity adjustment:


$$BV^{\left(t+1\right)}=C_{3}\cdot BV^{\left(t\right)}+C_{4}r_{2}(BP_{\text{best}}^{\left(t\right)}-BP^{\left(t\right)})+C_{5}r_{2}(BP_{\text{Gbest}}-BP^{\left(t\right)}),\;[6pt]BP^{\left(t+1\right)}=BP^{\left(t\right)}+BV^{\left(t+1\right)},$$

Where $r_{2}\sim 𝒰(0,1)$, and *C*_3_, *C*_4_, *C*_5_ are fixed coefficients controlling inertia and acceleration toward both local and global optima.

DTO offers rapid convergence and efficient local search due to its dynamic modulation of position and velocity parameters. However, like many population-based methods, it may exhibit premature convergence in highly multimodal landscapes. This limitation motivates its hybridization with the PRS strategy to enhance global search diversity.

#### 3.4.5 The PRS algorithm: Polar rose-inspired global search.

The Polar Rose Search (PRS) component is inspired by rose curves, a family of sinusoidal curves expressed in polar coordinates by the equation:


r(θ)=acos(kθ)orr(θ)=asin(kθ),

where *r* is the radial coordinate, θ is the polar angle, *a* is the amplitude (controlling the radius of the petals), and *k* is the angular frequency (determining the number and symmetry of petals). These curves create deterministic, symmetrical trajectories with high coverage of the angular domain, which is ideal for injecting structured diversity into population-based searches.

In PRS, candidate solutions are probabilistically perturbed along rose curves defined over polar projections of the search space. Each solution vector ${𝐱}_{i}\in {\mathbb{R}}^{d}$ is first transformed to a 2D polar representation by selecting a principal plane of variation (e.g., $\left(x_{i}^{\left(j\right)},x_{i}^{\left(j+1\right)}\right)$) and computing the corresponding polar coordinates (r,θ). The perturbed position is then obtained by modifying the radius:


$$r^{\prime }=a\cos(k\theta +\Delta \theta ),$$

Where Δθ is a rotational offset introduced to maintain temporal diversity, the updated Cartesian coordinates are retrieved using:


$$x_{i}^{\left(j\right)}=r^{\prime }\cos(\theta ),\;x_{i}^{\left(j+1\right)}=r^{\prime }\sin(\theta ).$$

The rose curves guarantee uniform angular distribution and controlled radial expansion/contraction, effectively balancing exploration and exploitation. The choice of *k* controls the complexity of the search pattern: odd values yield *k* petals, even values produce 2*k* petals, and irrational *k* leads to a non-repeating quasi-chaotic trajectory.

#### 3.4.6 DTO + PRS: Integrated hybrid framework.

The hybrid DTO+PRS algorithm synergistically combines the focused convergence dynamics of DTO with the global symmetry-driven diversification of PRS. The integration is orchestrated through an adaptive control mechanism that switches between DTO’s local update schemes and PRS-based perturbations based on an annealing-like schedule or entropy-based diversity metrics.

Specifically, the hybrid operates in two alternating phases:

1. **DTO Phase (Exploitation)**: Population updates follow the DTO’s swimming or flying equations, rapidly converging toward local optima. Feature selection is encoded as binary vectors, where continuous outputs are transformed using a sigmoid activation:
  $$S(x)=\frac{1}{1+e^{-10(x-0.5)}},$$
2. followed by thresholding at 0.5 to yield binary decisions.
3. **PRS Phase (Exploration)**: A subset of elite solutions is perturbed along polar rose trajectories. The polar parameters (*a*,*k*) are varied adaptively across iterations to avoid pattern saturation. This phase injects new candidate regions into the search process, improving escape from local minima and enabling re-diversification when population entropy drops below a critical threshold.

Algorithmically, the hybrid optimizer is formally defined as follows in Algorithm 1:

**Algorithm 1. Refined hybrid DTO+PRS algorithm.**



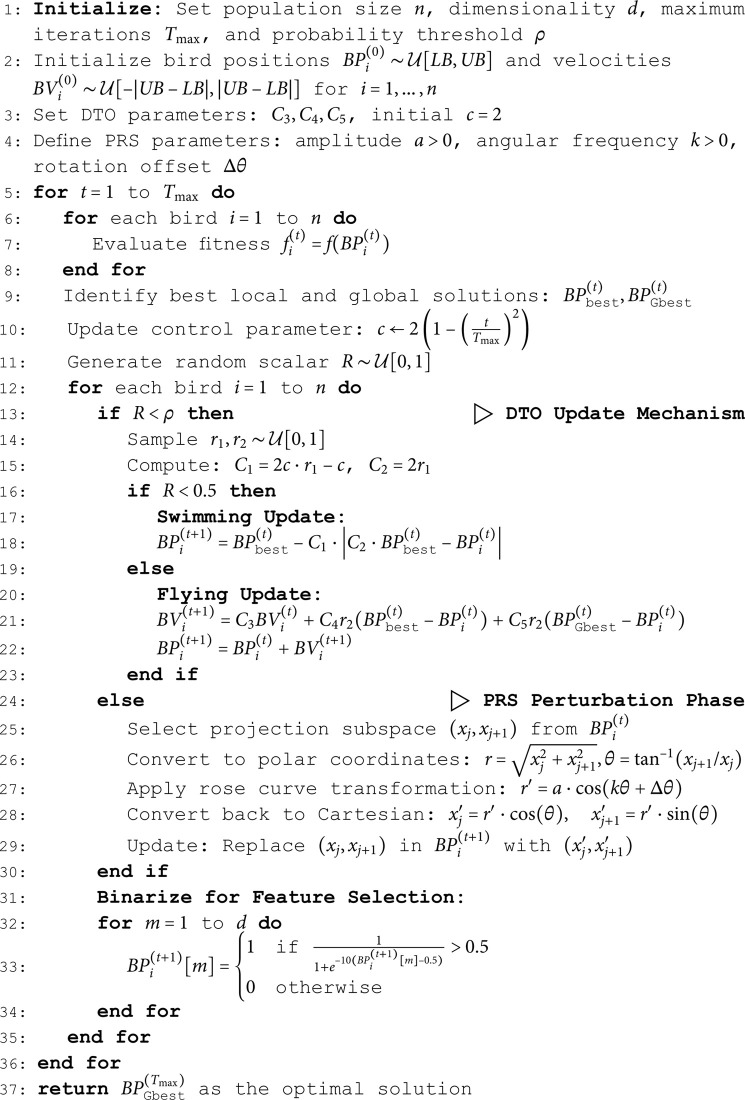



#### Theoretical justification and computational rationale.

Theoretical grounds demonstrate that DTO and PRS work together because their search-exploration methods support one another. The population distribution under DTO moves toward promising regions, using adaptive velocity and social interaction models for guidance. During its operation, PRS creates geometric diversity structures that help prevent premature convergence and allow the search to discover new attraction basins. Luxurious symmetrical sampling patterns within the polar curve system offer organized exhaustive search capabilities that reduce uncertainty in basic stochastic optimization processes.

The hybrid system lets users control the convergence behavior through angular frequency *k* and amplitude parameters *a* that can undergo real-time tuning or evolution. The adjustable parameters give the optimizer the power to solve problems with different shapes and requirement specifications.

As shown in Table 3, all metaheuristic algorithms were configured using standardized population sizes and iteration limits to ensure a fair and consistent benchmarking environment. This uniformity guarantees that any observed performance differences stem from algorithmic capabilities rather than configuration biases. While the proposed hybrid metaheuristic-deep learning framework demonstrates superior performance, it is crucial to acknowledge its computational complexity and resource requirements. The training of deep learning models, especially when integrated with metaheuristic optimization for feature selection and hyperparameter tuning, can be computationally intensive. This demands significant processing power (CPU), memory (RAM), and often specialized hardware like Graphics Processing Units (GPUs) for efficient execution, as detailed in the Materials and Methods section. This computational demand can be a limiting factor in real-time applications or in resource-constrained agricultural environments where access to high-performance computing infrastructure may be limited for individual farmers. Future work will focus on optimizing the framework for deployment in edge computing devices or cloud-based solutions to mitigate these computational barriers and enhance practical applicability in diverse farming settings.

**Table 3:** Initial parameter values for all algorithms and algorithm-specific settings.

Algorithm	Parameter	Value
*Common to all algorithms*		
All Algorithms	Population size	30
	Number of iterations	100
	Number of runs	20
	Search domain	0, 1
GWO	*a*	Linearly from 2 to 0
PSO	Inertia ($W_{\max},\;W_{\min}$)	[0.9, 0.6]
	Acceleration constants ($C_{1},\;C_{2}$)	[2, 2]
BA	Frequency range ($f_{\min},\;f_{\max}$)	0, 100
	Loudness (*A*)	$A_{0}=1,\;A_{\min}=0$
	Pulse emission rate (*r*)	[0, 1]
	Loudness & pulse-rate variation (*a* = *c*)	0.9
	Random walk for local search	[–1, 1]
	Number of bats (*n*)	[10, 50]
WOA	Spiral shape parameter (*b*)	Linearly from 2 to 0
SBO	Parameters ($r_{2},\;r_{3},\;r_{4}$)	[0, 1]
FA	Wormhole existence probability	[0.2, 1]
	Step size	0.94
DTO	Exponential control parameter (*c*)	2 to 0
	Parameters (*C*_1_, *C*_2_)	Based on *c* and $r_{1}\in [0,1]$
	Parameters (*C*_3_, *C*_4_, *C*_5_)	Constants (unspecified)
	Random factors ($r_{1},\;r_{2}$, *R*)	[0, 1]

**Fig 6:**
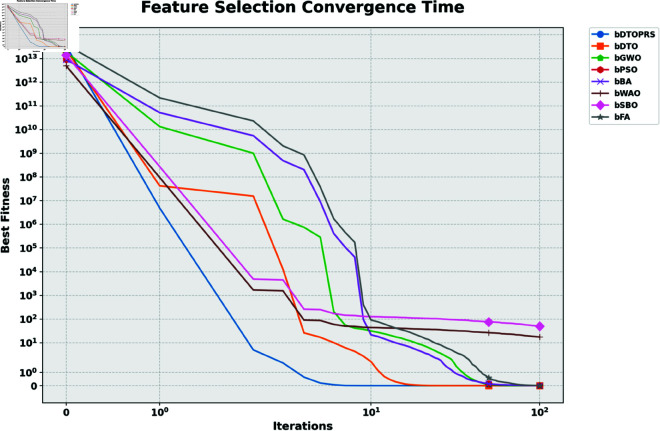
Classification convergence time showing fitness reduction over 100 iterations for DTOPRS-RBFN, DTO-RBFN, and benchmark models.

The convergence behavior of the classification models is shown in Fig 6, where DTOPRS-RBFN demonstrates a rapid and consistent drop in fitness over iterations, indicating superior convergence efficiency compared to DTO-RBFN, RBFN, LSTM, and RNN.

**Fig 7:**
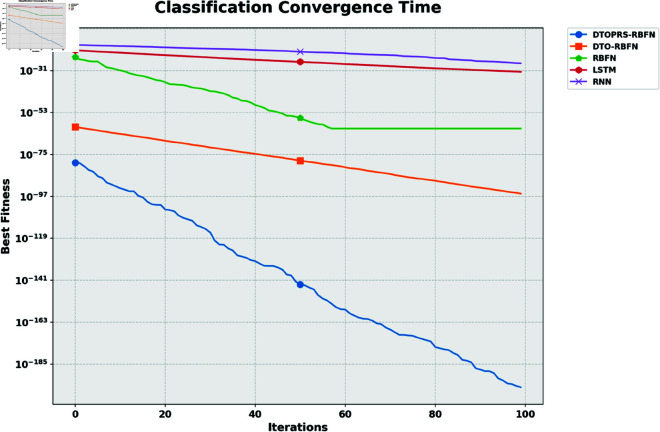
Feature selection convergence time comparison of various binary metaheuristic algorithms. bDTOPRS achieves rapid and stable convergence.

For feature selection, Fig 7 displays the convergence behavior across all tested metaheuristics. The bDTOPRS algorithm reaches optimal fitness values significantly faster than other methods, confirming its efficiency in identifying minimal and high-quality feature subsets.

The DTO+PRS hybrid metaheuristic is a strong, mathematically sound, performance-driven optimization solution. The ability of DTO + PRS to combine feature selection with optimization throughout a unified system makes it ideal for identifying optimal complex prediction models with high dimensions like water quality assessment in irrigation systems. The subsequent sections demonstrate the method’s superior empirical performance, which outcomes from the techniques explained in this section.

#### 3.4.7 SOTA benchmark optimization algorithms.

Evolutionary computation and nature-inspired search techniques have fully validated many metaheuristic algorithms for their competence in resolving complex optimization problems. This study employs six benchmark optimization algorithms as comparative baselines against the proposed hybrid approach (DTO + PRS). These algorithms are state-of-the-art (SOTA) in swarm intelligence and bio-inspired computation. This paper describes the operational behavior of multiple algorithms rather than showing their mathematical compositions because full computational details are omitted.

**Grey Wolf Optimizer (GWO):** Population-based optimization algorithm *Grey Wolf Optimizer (GWO)* operates through simulated natural behavior of grey wolves, including their hierarchical organization and hunting activities. The algorithm divides solutions into four hierarchical levels, from alpha down to beta and delta before omega, which dictates how the search direction will be managed. Position updates in the GWO follow wolf cooperative hunting methods by considering the influence of the three leading wolves in the pack [43]. GWO dynamically controls its exploration-exploitation balance, so it performs global searches initially until it changes to local refinement through progressive iterations. GWO finds widespread application because it offers basic implementation, stable convergence characteristics, and strong solution capabilities for continuous and discrete optimization.

**Particle Swarm Optimization (PSO):** The *Particle Swarm Optimization (PSO)* algorithm ranks among the first and most applicable swarm intelligence methods that came into existence due to observations of bird flocking and fish swarm behaviors. The population contains particles that function as potential solutions that modify their position and velocity through personal historical achievement and group-swarm-located excellence [44]. The independent forces of self and group components guide particles across the search space using exploratory and exploitative behavior. The prominent features of PSO include swift convergence speed with straightforward programming and overall flexibility; however, its performance deteriorates when dealing with highly multimodal functions.

**Bat Algorithm (BA):** The *Bat Algorithm (BA)* based its operational method on microbat echolocation techniques, where these flying mammals use sonar pulses to locate prey while navigating in darkness. When investigating the search environment, virtual bats in the population modify their locations, movement rates, emitted sound frequencies [45], and volume characteristics. Bats with echolocation adaptations utilize this system to manage the merger between exploration and exploitation activities based on their proximity to finding optimal solutions. The probabilistic frequency and loudness modification method fits BA exceptionally well when searching complicated non-convex search areas.

**Whale Optimization Algorithm (WOA):** The *Whale Optimization Algorithm (WOA)* uses inspiration from humpback whale bubble-net feeding behaviors to drive its operations. The bubble-net hunting method of whales generates trapping spirals that capture fish schools, and this approach uses logarithmic spiral mathematical models in the algorithm design. The significant components of WOA include prey-encircling behavior combined with spiraling movement through space and erratic prey-search movements [46]. WOA implements probabilistic mechanisms to balance its optimization techniques as it selects behaviors at random. This selection process produces a proper equilibrium between diversification and intensification. The adaptive movement capabilities of WOA demonstrate high performance in solving real-valued and combinatorial optimization problems.

**Satin Bowerbird Optimizer (SBO):** Through its mechanism, The *Satin Bowerbird Optimizer (SBO)* replicates the mating behaviors of satin bowerbirds that demonstrate bower construction coupled with intricate bower decoration to draw mates [47]. SBO features agents that model male birds that construct bower solutions before evaluation by visual appearance alongside novelty considerations. The optimization process combines exploiting elite solutions with exploration achieved through bower modifications. By modeling the competitive aspects of mating rituals, SBO enhances the convergence of its high-quality solutions when searching complex solution spaces.

**Firefly Algorithm (FA):** The *Firefly Algorithm (FA)* functions through a model derived from firefly communication using bioluminescence to create mating and prey attraction patterns. A key factor in FA’s operation includes using attractiveness based on objective function values (brightness) that result in fewer bright fireflies seeking out brighter ones [48]. The algorithm decreases in appeal as fireflies move farther from each other but maintain diverse searching capabilities at short and long distances. The simple rules of FA and its automatic organization capabilities allow it to solve rugged optimization problems effectively when dealing with high-dimensional features and multiple optimization scenarios.

The diverse analytic toolbox comprises six benchmark algorithms derived from different behavioral and biological foundations. A complete performance assessment of the proposed DTO+PRS hybrid method depends on its integration with these standard benchmark algorithms.

#### 3.4.8 Algorithm’s parameters setup.

Establishing a standardized and well-defined parameter space ensures the fairness and reproducibility of comparative evaluations across different metaheuristics. The initialization parameters for each optimization algorithm used throughout this study are summarized in Table 3, including shared values and specific tuning constants as applicable. These parameters were selected based on established practices in the literature and calibrated to balance convergence speed, solution diversity, and computational efficiency. A detailed explanation of each parameter is provided below:

**Common parameters:** The population size refers to the number of individual candidate solutions (agents, particles, wolves, bats, etc.) that constitute the optimization algorithm’s population. Generally, a larger population increases exploration capabilities but also raises computational cost. The number of iterations defines the maximum number of generations or cycles the optimization algorithm will run, determining the total search effort. The number of runs indicates how many independent executions of each algorithm are performed; multiple runs are crucial for statistical analysis to accommodate the stochastic nature of metaheuristics and ensure result robustness. The search domain specifies the boundaries within which candidate solutions can exist; for binary feature selection, this is typically [0, 1], representing feature selection or non-selection.

**Grey Wolf Optimizer (GWO) parameters:** The control parameter *a* decreases linearly from 2 to 0 over the course of iterations. This parameter is vital for managing the balance between exploration and exploitation: a higher value promotes broad searching, while a lower value encourages local refinement of solutions.

**Particle Swarm Optimization (PSO) parameters:** The inertia weight *W* (defined by $W_{\max}$ and $W_{\min}$) controls the influence of a particle’s previous velocity on its current velocity, with higher values favoring exploration and lower values promoting exploitation. The acceleration constants *C*_1_ and *C*_2_ serve as cognitive and social coefficients: *C*_1_ pulls particles toward their personal best positions, while *C*_2_ draws them toward the global best position found by any particle, regulating the maximum step size toward these optima.

**Bat Algorithm (BA) parameters:** The frequency range $\left(f_{\min},f_{\max}\right)$ defines the minimum and maximum echolocation frequencies used by bats, adjusted dynamically to control search behavior. Loudness *A* represents the intensity of emitted sounds, typically decreasing as a bat nears a promising solution to indicate exploitation; initial and minimum loudness values are denoted *A*_0_ and $A_{\min}$, respectively. The pulse emission rate *r* governs how often bats emit pulses, generally increasing during convergence, signaling a local search shift. The constants *a* = *c* control the updating of loudness and pulse emission rates. A random walk component with a range [–1,1] supports local search by perturbing bat positions. The population size specifically for BA is given by the number of bats *n*.

**Whale Optimization Algorithm (WOA) parameters:** The spiral shape parameter *b* defines the logarithmic spiral’s tightness during the exploitation phase, influencing how whales encircle their prey.

**Salp Swarm Algorithm (SBO) parameters:** Parameters *r*_2_, *r*_3_, and *r*_4_ are random numbers drawn from a uniform distribution [0,1]. These stochastic factors model the salps’ movement, affecting exploration and exploitation behaviors.

**Firefly Algorithm (FA) parameters:** The wormhole existence probability is a parameter that potentially enables global exploration or escape from local optima by allowing long-distance jumps via “wormholes." The step size regulates the magnitude of movement toward brighter fireflies, where larger values favor exploration and smaller values facilitate exploitation.

**Dipper Throated Optimization (DTO) parameters:** The exponential control parameter *c* typically decreases from a higher value (e.g., 2) to a lower value (e.g., 0) over iterations, balancing exploration and exploitation phases similarly to GWO’s parameter *a*. Parameters *C*_1_ and *C*_2_ guide dipper-throated birds’ movement toward the best solution, derived from *c* and a random variable $r_{1}\in [0,1]$, affecting step size and direction. Constants *C*_3_, *C*_4_, and *C*_5_ model specific behavioral aspects during different search phases (such as diving or swimming), though their exact roles depend on the DTO formulation. Random factors *r*_1_, *r*_2_, and *R*, drawn uniformly from [0,1], introduce stochasticity, enhancing exploration and preventing premature convergence.

### 3.5 Evaluation metrics

A strong evaluation method to analyze machine learning models together with optimization algorithms demands performance metrics that are both statistically sound, domain-specific, and robust. The study divides its assessment into two main categories: ML model classification capabilities (i) and solution quality (ii) obtained through metaheuristic feature selection processes.

#### 3.5.1 Machine learning prediction metrics.

As shown in Table 4, Multiple well-recognized metrics help evaluate the effectiveness of the ML models when used for irrigation water suitability classification. These assessment tools give a detailed picture of model discrimination power, the distribution of errors, and the capability to work with imbalanced classes. The set of metrics used for evaluation includes Accuracy combined with Sensitivity (also known as True Positive Rate, TPR) with Specificity (True Negative Rate, TNR), Positive Predictive Value (PPV) and Negative Predictive Value (NPV) along with the F-score.

**Table 4:** Machine learning prediction metrics and their equations.

Metric	Equation
Accuracy	$\frac{TP+TN}{TP+TN+FP+FN}$
Sensitivity (TPR)	$\frac{TP}{TP+FN}$
Specificity (TNR)	$\frac{TN}{TN+FP}$
Positive Predictive Value (PPV)	$\frac{TP}{TP+FP}$
Negative Predictive Value (NPV)	$\frac{TN}{TN+FN}$
F-score	$\frac{2\cdot TP}{2\cdot TP+FP+FN}$

Accuracy indicates the total model accuracy, while Sensitivity specifies correct predictions of positive cases, and Specificity measures correct negative classifications. PPV, along with NPV, strengthens the analysis of predictive class reliability. The F-score aligns precision and recall through an optimal balance between the two values. These metrics provide an all-encompassing assessment of model output behavior across data frequency patterns and prediction errors.

In the above equations, *TP*, *TN*, *FP*, and *FN* refer to the number of true positives, true negatives, false positives, and false negatives, respectively. These values are derived from the confusion matrix generated during model validation.

#### 3.5.2 Feature selection metrics.

The efficient operation of the ML pipeline requires feature selection as a fundamental step, particularly in analyzing high-dimensional environmental datasets, including water quality measurements. Feature selection algorithm assessments require both an evaluation of predictive model accuracy and a measurement of the optimization procedure.

As shown in Table 5, Average Selection Size and the set of metrics, including Average Error, serve as evaluation elements for this specific purpose. These metrics help measure classification error, model compactness, and evaluate fitness quality using Average, Best, Worst, and Standard Deviation of Fitness. The multiple metrics measure the consistency and robustness alongside the efficiency performance of the metaheuristic algorithm across various runs.

**Table 5:** Feature selection metrics and their definitions.

Metric	Equation or Description
Average Error	$\frac{1}{M}\sum_{i=1}^{M}{\text{Error}}_{i}$
Average Selection Size	$\frac{1}{M}\sum_{i=1}^{M}\frac{\left|{\text{Selected Features}}_{i}\right|}{\left|TotalFeatures\right|}$
Average Fitness	$\frac{1}{M}\sum_{i=1}^{M}f_{i}$
Best Fitness	$\min_{i=1,\ldots ,M}f_{i}$
Worst Fitness	$\max_{i=1,\ldots ,M}f_{i}$
Standard Deviation of Fitness	$\sqrt{\frac{1}{M-1}\sum_{i=1}^{M}(f_{i}-\bar{f}{)}^{2}}$

The table includes *M* independent optimization runs while *f*_*i*_ stands for the fitness value of the *i*-th run. The utilized fitness function combines classification error measurement with feature count evaluation through appropriate weight distribution.

Such integrated approaches for evaluating final model performance and selecting the best features provide rigorous transparency for measuring evaluation success.

## 4 Experimental results

The research conducts a comprehensive empirical study to evaluate the proposed hybrid optimization approach (*DTO+PRS*) versus many prevalent metaheuristic techniques and machine learning classification methods. A three-stage analysis evaluates how different optimization algorithms perform at feature selection, how chosen features affect ML model prediction, and includes a dedicated section for assessing the impact of optimization.

### 4.1 Feature selection results

To evaluate the effectiveness of the proposed bDTOPRS method for dimensionality reduction, a comparative performance analysis was conducted against seven alternative optimization algorithms. Table 6 presents the average classification error, selection size, fitness statistics, and time consumption for each method. The binary version of the hybrid DTO+PRS algorithm (bDTOPRS) achieved the lowest average classification error and selection size, confirming its superior capability to identify compact and highly informative subsets of features.

**Table 6:** Feature selection results.

	bDTOPRS	bDTO	bGWO	bPSO	bBA	bWAO	bSBO	bFA
Average error	0.6548	0.6792	0.7413	0.7358	0.7454	0.7356	0.7441	0.7342
Average Select size	0.6376	0.8376	0.9709	0.8376	0.9770	0.9001	0.9008	0.8721
Average Fitness	0.7480	0.7642	0.7725	0.7626	0.7855	0.7704	0.8023	0.8145
Best Fitness	0.6498	0.6845	0.7260	0.7429	0.6752	0.7345	0.7454	0.7332
Worst Fitness	0.7483	0.7514	0.8360	0.8106	0.7768	0.8106	0.8251	0.8308
Std. dev. Fitness	0.5703	0.5750	0.5932	0.5744	0.5843	0.5766	0.6353	0.6112
Time (s)	15.880	21.334	25.350	21.775	26.932	25.779	23.910	22.998

The results from Table 6 clearly demonstrate that bDTOPRS yields the most efficient and accurate feature subset. Its ability to achieve minimal classification error (0.6548) while selecting fewer features (63.76% of the total) shows its dominance in balancing accuracy and simplicity. Furthermore, the method exhibits lower variance in performance, as seen in the lowest standard deviation of fitness values, emphasizing its robustness and stability across repeated runs.

To validate the statistical significance of the observed differences across the tested algorithms, an ANOVA test was performed. The results in Table 7 demonstrate a highly significant difference among algorithms (*P*<0.0001), substantiating the robustness of bDTOPRS in feature selection.

**Table 7:** ANOVA test for feature selection.

ANOVA Table	SS	DF	MS	F (DFn, DFd)/P value
Treatment (between columns)	0.08163	7	0.01166	F(7, 72) = 111.5, *P*<0.0001
Residual (within columns)	0.007528	72	0.0001046	
Total	0.08916	79		

As illustrated by the ANOVA results in Table 7, the F-statistic confirms significant performance disparities between the tested algorithms. The high F-value (111.5) and extremely low p-value confirm that these differences are not due to random variation, reinforcing the comparative advantage of bDTOPRS.

Further, a Wilcoxon signed-rank test (Table 8) affirms that bDTOPRS significantly outperformed each comparator method with *P* = 0.002, highlighting its consistent statistical superiority.

**Table 8:** Wilcoxon test results for feature selection.

	bDTOPRS	bDTO	bGWO	bPSO	bBA	bWAO	bSBO	bFA
Theoretical median	0	0	0	0	0	0	0	0
Actual median	0.6548	0.6792	0.7413	0.7358	0.7454	0.7356	0.7441	0.7342
Number of values	10	10	10	10	10	10	10	10
Sum of signed ranks (W)	55	55	55	55	55	55	55	55
Sum of positive ranks	55	55	55	55	55	55	55	55
Sum of negative ranks	0	0	0	0	0	0	0	0
P value (two tailed)	0.002	0.002	0.002	0.002	0.002	0.002	0.002	0.002
Exact or estimate?	Exact	Exact	Exact	Exact	Exact	Exact	Exact	Exact
P value summary	**	**	**	**	**	**	**	**
Significant (α=0.05)?	Yes	Yes	Yes	Yes	Yes	Yes	Yes	Yes
Discrepancy	0.6548	0.6792	0.7413	0.7358	0.7454	0.7356	0.7441	0.7342

The Wilcoxon test in Table 8 provides non-parametric support for the superior performance of bDTOPRS. With all ranks positive and identical *P*-values below the 0.05 significance threshold, the test confirms that bDTOPRS consistently outperforms other optimizers across all trials.

### 4.2 Classification performance after feature selection

After optimal subsets of features were determined using bDTOPRS, various machine learning and deep learning classifiers were applied to assess prediction performance. As shown in Table 9, the RBFN model achieved the highest classification accuracy (94.21%), followed by LSTM (92.41%) and RNN (91.08%). These results confirm that feature selection via bDTOPRS facilitates improved learning and generalization across model architectures.

**Table 9:** Classification performance of ML and DL models.

Model	Accuracy	Sensitivity	Specificity	PPV	NPV	FScore	Time (s)
Support Vector Machine	0.8284	0.7986	0.8711	0.7511	0.8457	0.8988	508.45
Decision Tree	0.8449	0.8277	0.8711	0.7682	0.8657	0.9074	435.57
Random Forest	0.8545	0.8277	0.8945	0.7773	0.8719	0.9211	398.76
RNN	0.9108	0.8972	0.9283	0.8759	0.9187	0.9412	212.97
LSTM	0.9241	0.9061	0.9430	0.9058	0.9243	0.9432	201.63
RBFN	0.9421	0.9306	0.9543	0.9288	0.9429	0.9555	178.91

Discussion of Table 9 reveals that models trained on bDTOPRS-selected features achieve superior performance compared to those using the full feature set. RBFN’s accuracy lead validates its suitability for non-linear agricultural datasets, while the notable performance of LSTM and RNN shows the benefit of deep learning in sequence-aware or multi-relational input scenarios. Traditional models like SVM, DT, and RF lagged in performance, confirming the effectiveness of the metaheuristic-enhanced deep learning strategy.

**Fig 8:**
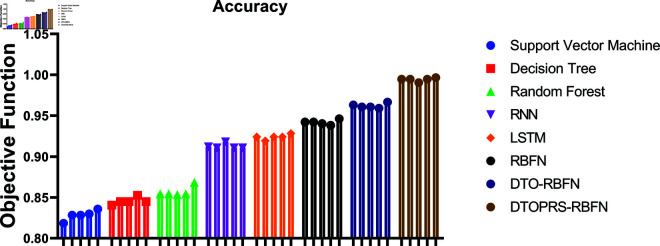
Comparison of classification accuracy across different machine learning models using the objective function metric. Models include traditional, deep learning, and hybrid RBFN-based methods.

Performance analysis of machine learning model classification skills on water quality data was executed through an accuracy comparison process. The research presents the accuracy results from eight methods shown in Fig 8. This comparison includes traditional models, including SVM, DT, Random Forest, deep learning models RNN and LSTM, and advanced variants RBFN, DTO-RBFN, and DTOPRS-RBFN. The objective function is displayed through the vertical axis since accuracy serves as the measurement. A visual display demonstrates how the hybrid model DTOPRS-RBFN outperforms traditional models, especially in race situations. Fig 8 provides a direct visual comparison of the peak classification accuracy achieved by each model. It clearly illustrates that the proposed hybrid DTOPRS-RBFN model consistently achieves the highest accuracy among all evaluated algorithms, suggesting its superior capability in optimizing the RBFN architecture for water quality classification. The significant gap between DTOPRS-RBFN and other models, including standalone RBFN, DTO-RBFN, and traditional machine learning and deep learning approaches, indicates the effectiveness of integrating Polar Rose Search with Dipper Throated Optimization for enhanced model optimization and feature selection. This figure serves as a primary indicator of the method’s classification prowess.

**Fig 9:**
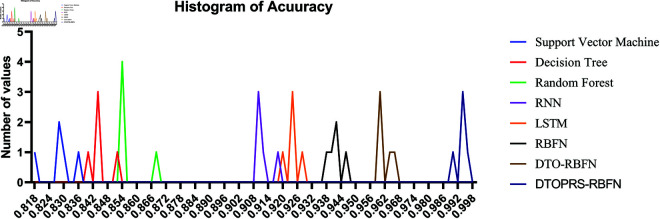
Histogram of accuracy values for different machine learning models across multiple runs. Each color and marker style represents a distinct model type.

Each classifier obtains additional reliability examination through accuracy value distributions illustrated in Fig 9. The illustration demonstrates the accuracy score disparities experienced by the Support Vector Machine and Decision Tree along with Random Forest and RNN LSTM , RBFN , DTOPRS-RBFN, and DTO-RBFN. The analysis of value frequency distribution allows us to understand average performance stability while determining models that demonstrate reliability through their ability to resist execution fluctuations. Fig 9 complements the accuracy comparison by visualizing the distribution of accuracy values obtained over multiple independent runs for each model. This histogram provides crucial insights into the robustness and stability of each classifier, beyond just their peak performance. A tighter, more concentrated distribution around a high accuracy value, as observed for DTOPRS-RBFN, signifies greater consistency and reliability. Conversely, models with wider or more dispersed distributions (e.g., SVM, DT) indicate higher variability in their performance across different runs, suggesting less stability. This visualization helps to identify models that are not only accurate but also consistently perform well, making them more dependable for practical agricultural applications where predictable outcomes are essential.

### 4.3 Performance after optimization

The final experimental stage involved full-scale optimization using the proposed DTOPRS method, applied specifically to the top-performing RBFN model. Table 10 demonstrates that DTOPRS-RBFN attained an outstanding classification accuracy of 99.46%, substantially outperforming both the standalone DTO-RBFN (96.07%) and baseline RBFN (94.21%). This result highlights the synergistic impact of simultaneous feature selection and optimization under the hybrid metaheuristic framework.

**Table 10:** Classification performance after optimization.

Model	Accuracy	Sensitivity	Specificity	PPV	NPV	FScore	Time (s)
DTOPRS-RBFN	0.9946	0.9959	0.9931	0.9954	0.9949	0.9939	105.66
DTO-RBFN	0.9607	0.9471	0.9751	0.9456	0.9612	0.9758	125.76

The performance comparison in Table 10 confirms that integrating PRS with DTO significantly elevates the model’s ability to generalize and classify accurately. The remarkable improvement in both sensitivity (99.59%) and specificity (99.31%) indicates precise discrimination between suitability classes, vital for real-world agricultural decision-making. The shorter inference time further adds to its practical applicability in field-deployed systems.

**Fig 10:**
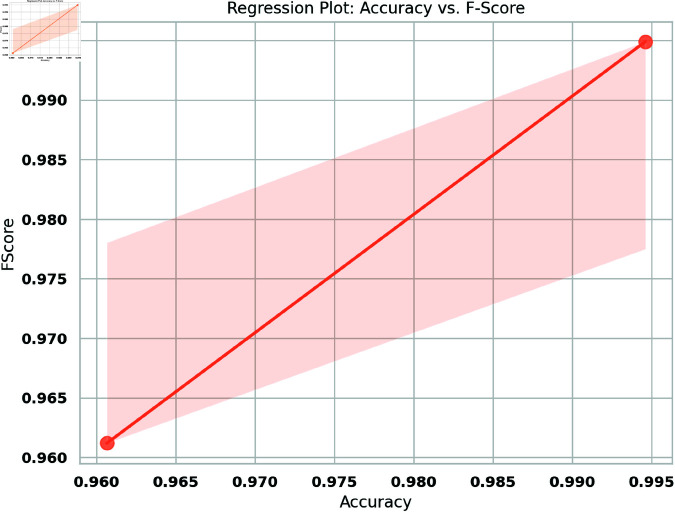
Regression plot showing the relationship between accuracy and F-score, with a fitted trend line and confidence interval.

A regression analysis determined the connection between the widely used metrics in classification problems—accuracy and F-score. The correlation between accuracy and F-score metrics shows a strong linear relationship as displayed through Fig 10. The detected relationship between accuracy and F-score indicates that precision-accurate predictive models often demonstrate superior performance in recall and precision measurement points.

**Fig 11:**
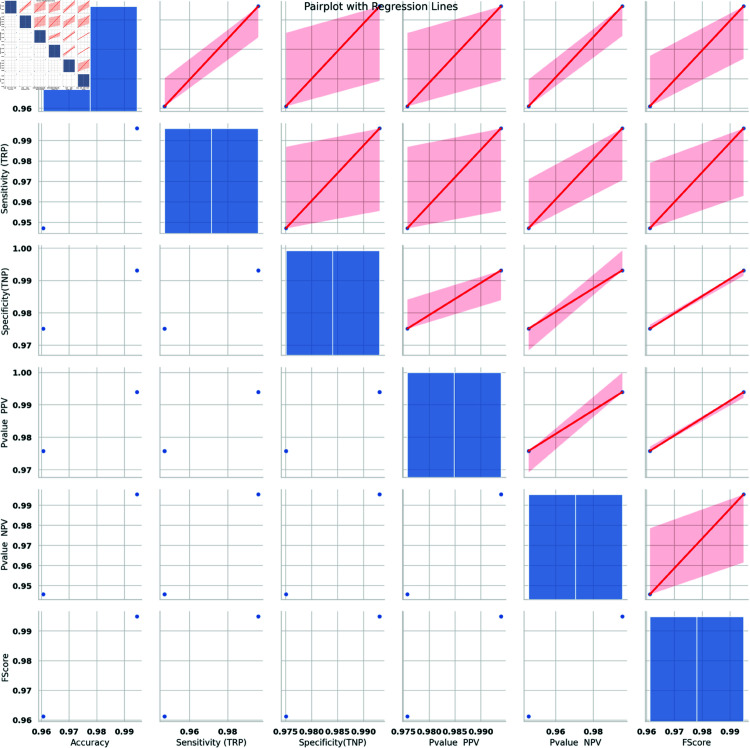
Pairplot of classification metrics including accuracy, sensitivity, specificity, PPV, NPV, and F-score with regression lines showing linear trends between metric pairs.

The relationships between crucial classification metrics were analyzed through a pair plot showing regression lines as illustrated in Fig 11. The visual presentation contains Accuracy

and Sensitivity (True Positive Rate) together with Specificity (True Negative Rate), Positive Predictive Value (PPV), Negative Predictive Value (NPV), and F-Score. Specifically, the diagonal panels show distribution data for each metric, while context diagrams with applicable regression lines contain off-diagonal panels. The pairplot presents a picture of metric relations, which helps researchers identify linear relationships and inter-metric dependencies.

To assess the statistical validity of the optimization improvements, an ANOVA test Table 11 confirmed significant differences among the optimized models (*P* < 0.0001).

**Table 11:** ANOVA test for optimization results.

ANOVA Table	SS	DF	MS	F (DFn, DFd)/P value
Treatment (between columns)	0.04251	4	0.01063	F(4, 45) = 183.4, *P* < 0.0001
Residual (within columns)	0.002608	45	0.00005795	
Total	0.04512	49		

As shown in Table 11, the ANOVA test yields a strong F-statistic (183.4), confirming statistically significant improvement in classification accuracy among the evaluated models. These results justify the use of DTOPRS as an effective optimization strategy in precision agriculture applications.

Additionally, a Wilcoxon signed-rank test (Table 12) revealed that DTOPRS-RBFN consistently outperformed DTO-RBFN, RBFN, LSTM, and RNN with statistical significance at α=0.05.

**Table 12:** Wilcoxon test results for optimization.

	DTOPRS-RBFN	DTO-RBFN	RBFN	LSTM	RNN
Theoretical median	0	0	0	0	0
Actual median	0.9946	0.9607	0.9421	0.9241	0.9108
Number of values	10	10	10	10	10
Sum of signed ranks (W)	55	55	55	55	55
Sum of positive ranks	55	55	55	55	55
Sum of negative ranks	0	0	0	0	0
P value (two tailed)	0.002	0.002	0.002	0.002	0.002
Exact or estimate?	Exact	Exact	Exact	Exact	Exact
P value summary	**	**	**	**	**
Significant (α=0.05)?	Yes	Yes	Yes	Yes	Yes
Discrepancy	0.9946	0.9607	0.9421	0.9241	0.9108

Table 12 further strengthens the conclusions from ANOVA, showing that DTOPRS-RBFN consistently ranks highest across ten repeated experiments. The exact *P*-value of 0.002 across all model comparisons highlights the robustness of the hybrid optimization approach.

## 5 Discussion

This study presents a hybrid optimization framework that integrates Dipper Throated Optimization (DTO) and Polar Rose Search (PRS) to enhance feature selection and optimization in predictive water quality modeling. The novel synergy between DTO’s local exploitation and PRS’s global exploration capabilities allows the model to overcome stagnation and escape local minima—two common pitfalls in traditional metaheuristics. DTO mimics the precise foraging dynamics of dipper birds, while PRS injects structured perturbations by tracing polar rose curves, offering systematic coverage of the solution space. This exploration–exploitation balance is dynamically controlled through an adaptive switching mechanism, enabling robust convergence across diverse optimization landscapes.

The strength of this approach is substantiated by extensive empirical evidence. Table 13 summarizes the statistical performance of all tested models, confirming that the proposed DTOPRS-RBFN architecture consistently outperforms conventional machine learning and deep learning classifiers. With a mean classification accuracy of 99.42% and the lowest standard deviation (0.0022), the model achieves both exceptional accuracy and reliability.

**Table 13:** Statistical summary of all models.

	SVM	Decision Tree	Random Forest	RNN	LSTM	RBFN	DTO-RBFN	DTOPRS-RBFN
Mean	0.8282	0.8455	0.8571	0.9125	0.9241	0.9418	0.9620	0.9942
Std. Deviation	0.0063	0.0043	0.0064	0.0031	0.0032	0.0030	0.0030	0.0022

The high accuracy of DTOPRS-RBFN (99.46%) is particularly impactful in the context of smart agriculture. In real-world irrigation systems, such precision enables better prediction of water suitability, ensuring that crops—especially water-intensive ones like potatoes—are not exposed to suboptimal irrigation sources. This can directly reduce resource waste, improve crop yields, and mitigate the risk of long-term soil degradation. In practice, even a marginal improvement in classification accuracy can translate to substantial agronomic and economic benefits at scale.

In terms of model generalization, multiple safeguards were implemented to prevent overfitting. These include the use of k-fold cross-validation during training, evaluation of prediction variance across multiple runs, and feature space reduction via bDTOPRS. The low standard deviation values observed in optimized configurations further confirm model stability. Still, generalization to novel environmental settings remains a critical concern for agricultural AI systems and warrants future field-level validation.

From a deployment perspective, the framework exhibits promising characteristics for real-time and embedded applications. The reduced number of features and low inference time (105.66 seconds for DTOPRS-RBFN) make it feasible for integration with edge computing platforms. This is critical in modern agricultural environments, where connectivity, power, and compute resources may be constrained. While full energy-efficiency profiling is outside the current study’s scope, the lean model architecture lays the groundwork for future sustainable deployments.

A key contribution of this work is the interpretability of both feature selection and optimization. Selected features—such as sulfate, chloramines, and turbidity—are not only statistically significant but also well-aligned with agronomic best practices. These indicators are commonly monitored in agricultural water management due to their known impact on soil chemistry and crop health. Similarly, optimized hyperparameters like the Gaussian kernel width and neuron counts offer meaningful insights for environmental model calibration. These interpretable components strengthen stakeholder trust and enhance transparency.

**Fig 12:**
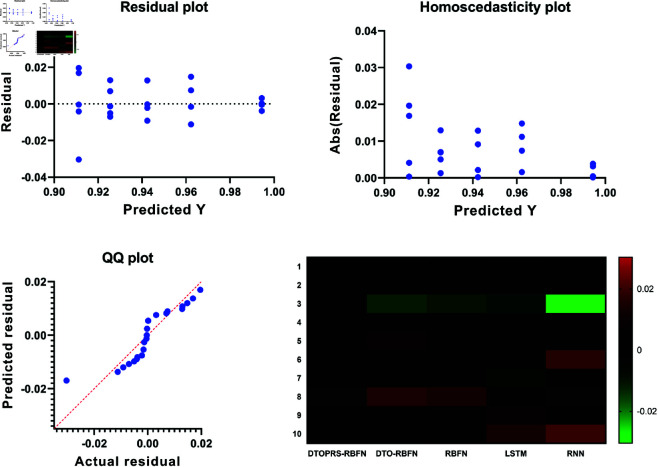
Comparative visual performance analysis across models using key classification metrics and their interrelationships.

Fig 12 offers a comparative visual breakdown of prediction metrics across models, including accuracy, sensitivity, specificity, and predictive values. The clear visual clustering of DTOPRS-RBFN near optimality further reinforces its dominance across evaluation metrics.

The internal structure of the DTO+PRS pipeline deserves emphasis. The two algorithms are not applied in isolation or sequentially but operate in an intertwined, iterative fashion. DTO provides localized refinements, while PRS introduces global search diversity through periodic perturbations based on polar rose curves. This interaction is governed by entropy-based triggers and annealing schedules to maintain balance. Convergence is assessed through population entropy and fitness plateaus rather than fixed thresholds, ensuring adaptive stopping.

Despite its strengths, several limitations merit discussion. The model currently assumes a relatively clean dataset preprocessed via statistical imputation and normalization. However, agricultural sensor networks often suffer from frequent signal loss, drift, or anomalies. A next step would be to integrate robust anomaly detection and noise-tolerant modeling directly into the learning pipeline. Moreover, although the proposed method was evaluated on a real dataset, it has not yet been deployed in a field environment. Offline validation, while valuable, cannot fully capture the variability and operational challenges of real-world agriculture.

**Fig 13:**
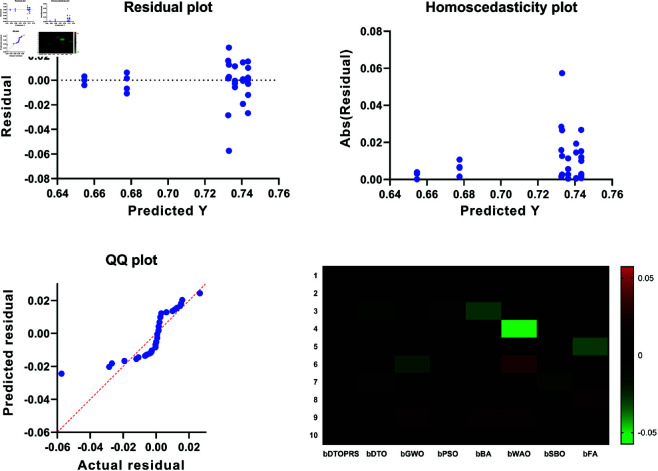
Feature selection diagnostics: distribution, correlation, and contribution heatmaps from the bDTOPRS process.

Fig 13 provides further support for the interpretability and consistency of the feature selection process by visualizing selection frequencies and their statistical contributions. It confirms that bDTOPRS repeatedly converges on environmentally meaningful features across trials.

In terms of cost-effectiveness, future work should benchmark the model’s energy and hardware requirements against practical constraints of rural deployments. Collaborations with agronomists and irrigation engineers would also help translate prediction outputs into actionable watering protocols or alerts. Finally, expanding the approach to cover additional crops, soil types, and sensor modalities would significantly improve its versatility and impact.

In conclusion, the proposed DTO+PRS framework offers a mathematically grounded, biologically inspired, and agriculturally relevant solution for precision water quality classification. By merging deep learning with dynamic metaheuristic optimization, the model delivers not only superior accuracy but also improved interpretability, efficiency, and field readiness. These qualities make it a strong candidate for inclusion in next-generation smart irrigation and sustainable farming systems.

## 6 Conclusion and future work

This study introduced a novel hybrid optimization framework that synergistically combines Dipper Throated Optimization (DTO) and Polar Rose Search (PRS) to enhance deep learning model performance for precision agriculture applications. By effectively addressing both the challenges of high-dimensional feature spaces and the sensitivity of hyperparameters, the proposed approach enables the development of highly accurate, stable, and interpretable predictive models for irrigation water quality assessment in potato cultivation. The integration of bio-inspired behavioral strategies with mathematically structured exploration mechanisms proved essential for achieving these advancements.

Beyond demonstrating superior classification accuracy, the findings underscore the model’s potential to inform real-world agricultural decisions that promote sustainable water management, optimize resource utilization, and ultimately improve crop health and yield stability. The ability to reduce input dimensionality while maintaining robustness enhances model deployment feasibility in diverse farming environments with varying data availability and computational constraints.

Despite the promising results, this study acknowledges several limitations that offer critical directions for future research. Firstly, the current model’s performance is inherently dependent on the quality and availability of high-quality sensor or laboratory-derived water quality data. Any inaccuracies or inconsistencies in the input data could impact the reliability of the predictions. Secondly, the generalizability of the findings across diverse geographies and various crop types, beyond potato cultivation, remains to be fully established. The reliance on a single dataset and specific experimental conditions, while valuable for initial validation, limits the immediate assertion of universal applicability. Sustainable farming practices necessitate adaptable solutions that perform consistently across different regions and water sources, yet the study does not fully demonstrate scalability or reproducibility in varied agricultural settings. Thirdly, while the framework enhances efficiency, the computational demand for real-time applications, particularly in resource-constrained environments, requires further optimization and hardware considerations.

Looking ahead, this framework opens several promising avenues for further research and practical implementation. Validation on more diverse datasets covering multiple crops and environmental conditions is critical to establish broader applicability and resilience. This includes conducting extensive field trials in varied agricultural settings to demonstrate the practical scalability and reproducibility of the proposed solution. Incorporating real-time data streams from IoT-enabled sensors, combined with weather and remote sensing inputs, could facilitate adaptive irrigation management systems responsive to dynamic field conditions. Furthermore, embedding explainable AI techniques would enhance transparency, build stakeholder trust, and assist agronomists in interpreting model outputs for informed decision-making. A more comprehensive comparative evaluation, including rigorous benchmarking against an even wider array of state-of-the-art machine learning and deep learning models, would further strengthen the claims of superiority and practical relevance.

Additionally, exploring the transferability of the DTO+PRS hybrid optimizer to related agricultural domains—such as soil nutrient profiling, pest risk prediction, and climate-adaptive planting strategies—could establish this approach as a foundational component of future agri-intelligence platforms. As the agriculture sector increasingly embraces digital transformation, the marriage of intelligent optimization with deep learning, as demonstrated here, offers a scalable, efficient, and scientifically grounded pathway toward sustainable farming and food security.
